# Susceptibility to online misinformation: A systematic meta-analysis of demographic and psychological factors

**DOI:** 10.1073/pnas.2409329121

**Published:** 2024-11-12

**Authors:** Mubashir Sultan, Alan N. Tump, Nina Ehmann, Philipp Lorenz-Spreen, Ralph Hertwig, Anton Gollwitzer, Ralf H. J. M. Kurvers

**Affiliations:** ^a^Center for Adaptive Rationality, Max Planck Institute for Human Development, Berlin 14195, Germany; ^b^Department of Psychology, Humboldt University of Berlin, Berlin 12489, Germany; ^c^Exzellenzcluster Science of Intelligence, Technical University of Berlin, Berlin 10587, Germany; ^d^Department of Psychology, University of Konstanz, Konstanz 78457, Germany; ^e^Center Synergy of Systems and Center for Scalable Data Analytics and Artificial Intelligence, TUD Dresden University of Technology, Dresden 01069, Germany; ^f^Department of Leadership and Organizational Behaviour, BI Norwegian Business School, Oslo 0484, Norway

**Keywords:** misinformation, analytical thinking, partisan bias, illusory truth effect, signal detection theory

## Abstract

Nearly five billion people use and receive news through social media and there is widespread concern about the negative consequences of misinformation on social media (e.g., election interference, vaccine hesitancy). Despite a burgeoning body of research on misinformation, it remains largely unclear who is susceptible to misinformation and why. To address this, we conducted a systematic individual participant data meta-analysis covering 256,337 unique choices made by 11,561 US-based participants across 31 experiments. Our meta-analysis reveals the impact of key demographic and psychological factors on online misinformation veracity judgments. We also disentangle the ability to discern between true and false news (discrimination ability) from response bias, that is, the tendency to label news as either true (true-news bias) or false (false-news bias). Across all studies, participants were well above-chance accurate for both true (68.51%) and false (67.24%) news headlines. We find that older age, higher analytical thinking skills, and identifying as a Democrat are associated with higher discrimination ability. Additionally, older age and higher analytical thinking skills are associated with a false-news bias (caution). In contrast, ideological congruency (alignment of participants’ ideology with news), motivated reflection (higher analytical thinking skills being associated with a greater congruency effect), and self-reported familiarity with news are associated with a true-news bias (naïvety). We also find that experiments on MTurk show higher discrimination ability than those on Lucid. Displaying sources alongside news headlines is associated with improved discrimination ability, with Republicans benefiting more from source display. Our results provide critical insights that can help inform the design of targeted interventions.

## Significance

It remains unclear who is susceptible to misinformation. We synthesized 31 studies to uncover how key demographic and psychological factors impact misinformation susceptibility. We distinguished between the ability to differentiate true from false news (discrimination ability) and response bias: the tendency to label news as either true (true-news bias) or false (false-news bias). We found that older adults, Democrats (compared to Republicans), and those with higher analytical thinking skills show greater discrimination ability. Ideological congruency (alignment of participants’ ideology with news), motivated reflection (higher analytical thinking skills being associated with a greater congruency effect), and self-reported familiarity with news were linked with a true-news bias. Our results provide insights for better theory building and for designing targeted interventions.

Belief in misinformation can have far-reaching consequences—for example, in politics (e.g., the US Capitol riots; 1), health (e.g., vaccine hesitancy; 2), and climate change (e.g., resistance to climate-friendly behavior; 3). Despite the surge of research on misinformation, it is largely unclear who is susceptible to it and why. Previous studies typically investigate single demographic (e.g., age, political identity) or psychological factors (e.g., analytical thinking, ideological congruency) in isolation, often leading to conflicting results. These studies tend to employ the widely used news headline paradigm, where participants evaluate the veracity of news headlines (i.e., a headline, potentially accompanied by a byline, and/or an image). Pooling individual participant data from the news headline paradigm—totaling 256,337 unique choices made by 11,561 participants across 31 experiments—we conducted a systematic meta-analysis on how key demographic and psychological factors impact online misinformation veracity judgments. Understanding susceptibility to misinformation is crucial for identifying vulnerable groups, designing tailored interventions, and combatting its far-reaching consequences.

We examined four demographic factors that represent important population-level characteristics—age, gender, education, and political identity—and four psychological factors that are considered to play a pivotal role in judging misinformation—analytical thinking (4), ideological congruency (alignment of participants’ ideology with news; i.e., partisan bias; 5), motivated reflection (higher analytical thinking skills being associated with a greater congruency effect; 6), and self-reported familiarity with news (i.e., the illusory truth effect; 7). We also explored meta-questions on the effects of the topic of news headlines (e.g., political, health), the study platform (e.g., MTurk), and the source being displayed alongside news headlines. To do so, we conducted an individual participant data meta-analysis (i.e., using raw trial-level data; 8, 9) and applied signal detection theory (SDT; 10) to delineate how the above demographic and psychological factors influence two frequently confounded decision-making mechanisms: discrimination ability, that is, the ability to distinguish between true and false news, and response bias, that is, the tendency to classify news as true (true-news bias) or false (false-news bias). Despite being conflated in much of the literature, these mechanisms can have very different ramifications for theory and intervention building (e.g., individualized interventions; 11, 12).

Given our approach, this meta-analysis uniquely allows us to touch upon key debates in the literature: do older adults, despite their lower digital literacy and propensity to share false news, have more accurate veracity judgments compared to their younger counterparts? What is the effectiveness of formal education with its promises of critical thinking skills for navigating online information environments? Relatedly, what is the potential of analytical thinking skills to improve veracity judgments? What is the influence of political identity in shaping distinct perceptions of truth, including the role of ideological congruency? And, finally, how much does familiarity breed belief in misinformation? We discuss each demographic and psychological factor next.

On one end of the age spectrum are “digital natives”—younger generations who were born into the digital age. On the other end are older adults, who entered the digital world later in life and are less digitally literate (13, 14). This divide can manifest in the way people judge and share misinformation. The literature indicates that older adults visit more low-quality news websites and share more misinformation compared to younger adults (15, 16, 17). However, studies suggest that older adults are generally better (18, 19, 20), or as good as their younger counterparts, at judging the veracity of news (21). Our meta-analysis aims to provide clearer insights into this apparent paradox.

There appear to be gender-based differences in news engagement patterns. Compared to male participants, female participants consume less news (22), express lower interest in news (23), and are more likely to avoid news (23, 24). Nevertheless, the literature has produced mixed findings on the impact of gender on veracity judgments. Wolverton and Stevens (25) and Fadhila et al. (26) report a null effect for gender, whereas Halpern et al. (27) and Pennycook and Rand (20; although in only one of their two studies) find that female participants perform worse than male participants at judging the veracity of news headlines. Note, however, that Halpern et al. (27) focus only on false news, making it impossible to distinguish between discrimination ability and response bias. We aim to overcome some of these methodological inconsistencies and address the effect of gender on misinformation veracity judgments.

Formal education teaches people critical thinking skills—challenging assumptions, scrutinizing sources, and evaluating information. Multiple studies find that higher formal education correlates with better veracity judgments (18, 25). However, a parallel strand of research posits that traditional education in terms of critical thinking is insufficient for the digital age (28). Even people with high levels of education (e.g., professors) are swayed by factors such as objective-sounding language and official-looking websites (29, 30). The relationship between education and veracity judgments requires clarification.

Engagement with (mis)information online is heavily influenced by people’s political identities, as it can occur in echo chambers (formed by either algorithmic or user-created processes) where people are surrounded by like-minded peers (31, 32). Echo chambers and similar structures also play an important role in an ongoing debate over whether the information ecosystems of Democrats and Republicans diverge enough to drive polarization and shape distinct realities. Various studies report that Democrat participants outperform Republican participants in assessing news veracity (33, 34, 35, 36, 37). Our meta-analysis aims to delineate these potentially crucial asymmetries.

Analytical thinking is a concept rooted in dual-process theory (38). In the context of misinformation, it holds that people fail to discriminate between true and false news because they rely on intuitive, albeit erroneous, responses instead of taking a moment to deliberate. Past research has shown that higher analytical thinking skills are associated with better discrimination ability, and in some cases, an increased tendency to treat news as false (i.e., false-news bias; 4, 39, 40).

The concept of ideological congruency is based on ingroup versus outgroup favoritism, as outlined in social identity theory (5, 41). According to this account, people treat news content that is congruent (incongruent) with their political identity favorably (unfavorably). In the context of the news headline paradigm, news headlines tend to be pretested on whether they favor either Democrats or Republicans, allowing a measure of (in)congruency with participants’ political identities (Democrat, Republican). As a function of ideological congruency, people may treat false news as true because it is congruent with their political identity, or treat true news as false because it is incongruent with their political identity. Indeed, past studies have found that both Democrat and Republican participants are more likely to judge news headlines as true (false) when the headlines align (misalign) with their ideology (4, 39, 40).

Motivated reflection is an interplay between analytical thinking and ideological congruency (i.e., an interaction on the response bias). Individuals with higher analytical thinking skills, known to have higher discrimination ability and a more cautious approach, paradoxically exhibit greater susceptibility to ideological congruency (6, 42). They are suspected to use their higher analytical thinking skills to rationalize information that aligns (misaligns) to their ideological beliefs as true (false). The literature shows conflicting results for motivated reflection. For example, some (40) find an effect for motivated reflection on veracity judgments, whereas others (39) do not (see also refs. 33 and 43, 44, 45).

Familiarity with a news headline is thought to increase the fluency of information processing, and as such, may act as a meta-cognitive cue for accuracy (46, 47, 48). Because familiar news is easier to process, it is more likely to be perceived as true. This is a robust effect (for reviews, see refs. 46 and 49) across different levels of cognitive ability (50) and prior accurate knowledge (51; trivia statements), even months after exposure (52; trivia statements). Familiarity mostly impacts response bias but has also been associated with reduced discrimination ability (39, 40). All in all, with our meta-analysis, we seek to speak to the robustness of the findings related to the psychological factors and susceptibility to misinformation.

For all the factors above, the distinction between discrimination ability and response bias has been blurry. This is because most studies on misinformation focus on accuracy—summed across true and false news or for true and false news separately. Focusing on accuracy conflates participants’ ability to distinguish between true and false news with their general tendency to judge news as true or false (e.g., due to being cautious or naïve, or due to experimental demands). This is highly relevant for designing interventions to combat misinformation. For example, Modirrousta-Galian and Higham (53) found that an increase in participants’ ability to spot misinformation after an intervention was in fact attributable to a higher false-news bias (i.e., a general tendency to judge news as false), not higher discrimination ability (see also refs. 11, 12, and 39). Effective interventions also require an understanding of whether ideological congruency improves veracity judgments—that is, because people know more about ingroup issues—or whether it shifts people to a true-news bias. For these reasons, taking advantage of the framework of SDT is crucial, as it allows us to distinguish between discrimination ability and response bias.

Based on past results, we hypothesized higher discrimination ability for older adults, individuals with higher formal education, and Democrats, and no effect of gender. Due to the conflation of discrimination ability and response bias in previous studies, we had no specific hypotheses for these effects. Turning to the psychological factors, we hypothesized higher discrimination ability for individuals with higher analytical thinking skills and for unfamiliar news headlines. We also hypothesized a higher tendency to classify news headlines as true (true-news bias) when they align with one’s political identity (ideological congruency) and when they are familiar.

To test our hypotheses, we conducted a preregistered systematic individual participant data meta-analysis (https://osf.io/s2ejg/). All studies were US-based and used the experimental news headline paradigm, including a nonconfounded measure of veracity (i.e., no sharing decisions; for full eligibility criteria, see *Materials and Methods*). This allowed us to pool individual participant data spanning the eight demographic and psychological factors. An individual participant data meta-analysis (8, 9) allows for a more detailed analysis compared to an effect-size-based meta-analysis. Specifically, we were able to use trial-level raw data to conduct an SDT analysis using a single mixed-effects model across studies. This helped to reduce variation in the statistical methods across studies and enabled us to conduct subgroup analyses, consolidating the demographic and psychological factors into a unified model to assess their relative strengths. The mixed-effect model was also able to accommodate the hierarchical nature of the data, helping to account for risk of publication bias by better controlling participant-, study-, and headline-level variability. Overall, our approach provides a comprehensive explanation of how each factor is associated with judging the veracity of (mis)information.

## 1. Results

### 1.1. Study and Participant Characteristics.

Our search for articles using relevant search terms on databases and via mailing lists returned 4,666 results. Of these, 21 articles, encompassing 31 studies, were included in the final analysis (for details on the screening process, see *Materials and Methods*). In total, 256,337 unique veracity judgments (e.g., “true”/“false”) made by 11,561 participants (*M*_age_ = 41.29 y, SD=15.68,range = 18 to 88) were included, comprising 53.91% identifying as female (46.09% male) and 41.85% identifying as Republican (58.15% Democrat).

### 1.2. SDT, Demographic Factors, and Psychological Factors.

**Fig. 1.**
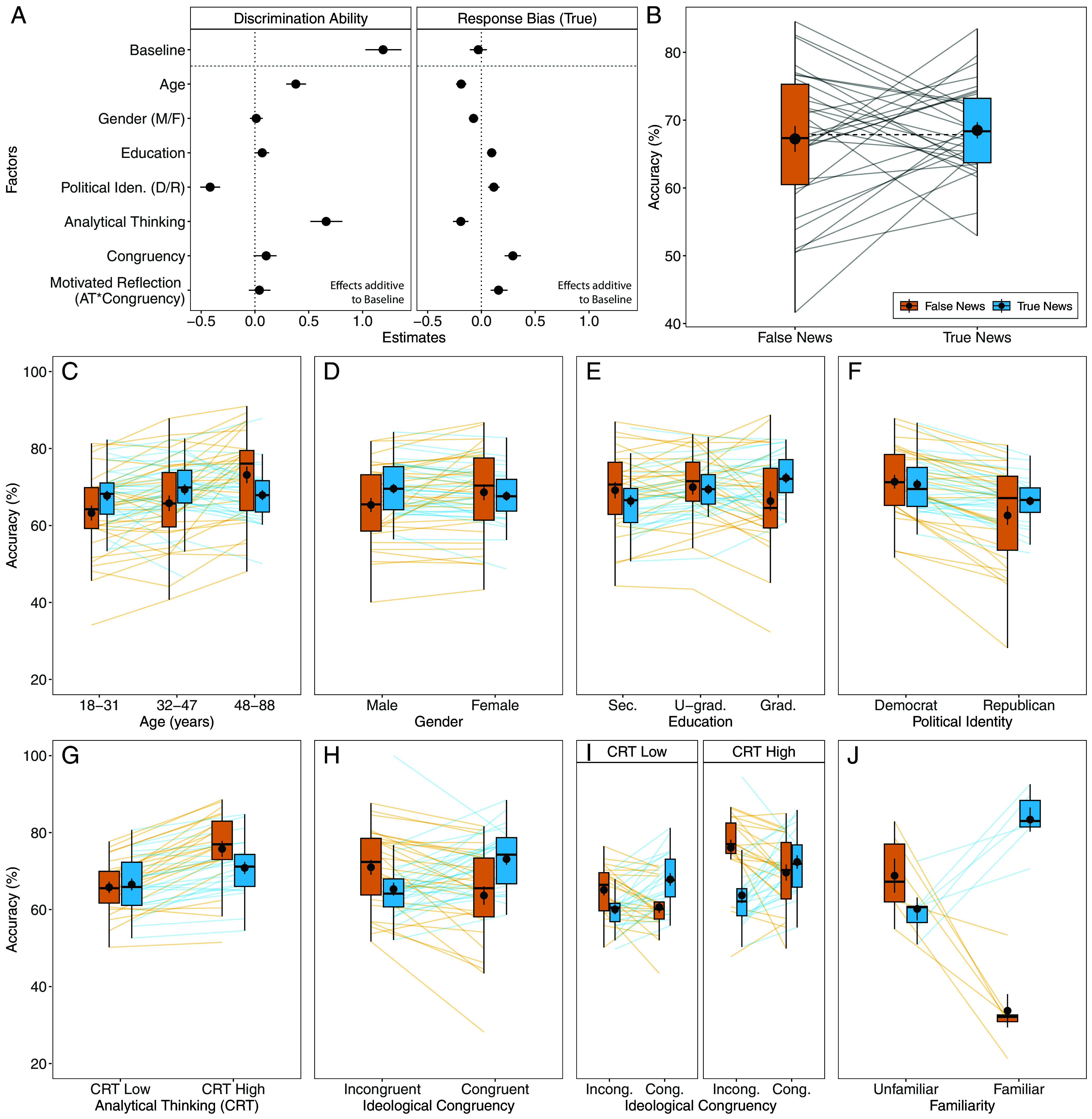
Signal detection analysis, true and false news accuracy, and true and false news accuracy across demographic and psychological factors. (*A*) SDT model estimates. All results derive from a single SDT analysis using participants’ responses (false news or true news) as the response variable but are shown in two panels for clarity. *Left*: Estimates for discrimination ability, with more positive (negative) values indicating higher (lower) discrimination ability. *Right*: Estimates for response bias, with more positive (negative) values indicating a higher likelihood to judge headlines as true (false). Baseline: Overall estimate of discrimination ability (*Left*) and response bias (*Right*). Gender (M/F) = coded Male to Female. Political Iden. (D/R) = political identity, coded Democrat to Republican. Congruency = ideological congruency. AT*Congruency = interaction between Analytical Thinking and Congruency. Dots represent the mean; error bars represent the 95% CI of the posterior distribution. All factors were mean centered. (*B*) Accuracy for true and false news headlines. Gray lines connect the mean accuracy scores for false and true news headlines within a study. Large dots represent the aggregate mean with SE; the dashed black line represents the aggregate mean across all studies. Boxplots show the median and the interquartile range (IQR); whiskers indicate an additional 1.5 IQR. (*C*–*F*) Accuracy for true and false news headlines across demographic factors. Age is split into tertiles for visualization purposes. Colored lines connect the accuracy scores within a study (e.g., false news accuracy across age tertiles). Sec = Secondary education. U-grad = undergraduate education. Grad = graduate education. (*G*–*J*) Accuracy for true and false news headlines across psychological factors. Analytical thinking measured via the cognitive reflection test (CRT) scores is median split for visualization purposes into CRT Low and CRT High. Incong: Incongruent. Cong: Congruent.

We conducted the SDT analysis using a Bayesian generalized linear mixed-effects model where all factors were mean centered (for more, see *Materials and Methods*). Regarding interpretation of the SDT model, we report baseline discrimination ability (response bias), which is the average discrimination ability (response bias; Fig. 1*A*). The effect of each factor should be interpreted in an additive manner to these effects, conditional on the other factors being at their zero-level (their means). Higher (lower) discrimination ability is indicated by more positive (negative) values (Fig. 1*A*). For response bias, a higher likelihood to judge headlines as true (false) is indicated by more positive (negative) values (Fig. 1*A*). A true-news response bias refers to a tendency to judge news as true; whereas, a false-news bias refers to a tendency to judge news as false. All else being equal, the former leads to higher accuracy for true news headlines and lower for false news headlines; whereas the latter leads to higher accuracy for false news but lower for true news.

Participants had a discrimination ability credibly higher than zero ($\beta_{\;\text{Discrimination Ability}}$ = 1.19, 95% credible intervals [CI] = 1.03 to 1.36; see baseline in Fig. 1*A*). This effect was robust across all studies (Fig. 1*B*; for separate estimates for all studies, see *SI Appendix*, Fig. S1*A*). This effect is descriptively visualized in Fig. 1*B*, showing that participants had better-than-chance overall accuracy (67.87%) across all studies and news headlines (dotted line). The baseline response bias was not credibly different from zero ($\beta_{\;\text{Response Bias}}$ = −0.03, CI = −0.11 to 0.05; see baseline in Fig. 1*A*). Corroborating this, Fig. 1*B* shows that participants were similarly accurate for true (68.51%) and false headlines (67.24%), as evidenced by the mean and median values in the boxplots. Although there was no overall effect of response bias, single studies varied widely in response bias (see individual study lines in Fig. 1*B*), showing a true bias, a false bias, or no bias (for separate estimates for all studies, see *SI Appendix*, Fig. S1*B*). In sum, averaged over all studies and factors, participants generally performed better-than-chance and did not have an overall response tendency to treat news as either true or false. We next turn to the results of the demographic and psychological factors.

#### 1.2.1. Age.

Age had a positive effect on discrimination ability (β = 0.38, CI = 0.29 to 0.47; Fig. 1*A*), indicating that older participants achieved higher overall accuracy (Fig. 1*C*). Moreover, we also found a negative and credible effect of age on response bias (β = −0.19, CI = −0.24 to −0.14]; Fig. 1*A*), suggesting that, with increasing age, individuals were generally more likely to judge a news headline as false. This resulted in older adults having higher accuracy for false news than true news (Fig. 1*C*; see *SI Appendix*, Fig. S2 *A* and *B* for these effects per study). Being older was thus associated with better discrimination ability and a higher overall false-news bias, which explains the widening gap in true and false news accuracy with higher age (Fig. 1*C*).

#### 1.2.2. Gender.

There was no credible effect of gender on discrimination ability (β = 0.01, CI = −0.05 to 0.07; Fig. 1*A*). We did, however, find a small, negative, and credible association between gender and response bias (β = −0.07, CI = −0.12 to −0.03; Fig. 1*A*), with female participants being more likely than male participants to classify news headlines as false. Female participants had slightly higher accuracy for false news headlines than male participants, who in turn had slightly higher accuracy for true news (Fig. 1*D*). *SI Appendix*, Fig. S2 *C* and *D* show these effects per study.

#### 1.2.3. Education.

Education did not have a credible effect on discrimination ability (β = 0.07, CI = 0.00 to 0.13; Fig. 1*A*). We found a small, positive, and credible effect of education on response bias (β = 0.1, CI = 0.05 to 0.14; Fig. 1*A*), with more educated individuals displaying a true-news bias that resulted in higher accuracy for true news and lower accuracy for false news (Fig. 1*E*). Having more years of formal education was thus associated with an increased tendency to view news as true. *SI Appendix*, Fig. S3 *A* and *B* show these effects per study.

#### 1.2.4. Political identity.

Political identity had a strong, credible, and negative effect on discrimination ability (β = −0.42, CI = −0.51 to −0.32; Fig. 1*A*), with Republican participants achieving lower overall accuracy compared to Democrat participants (Fig. 1*F*). It also had a small, positive, and credible effect on response bias (β = 0.12, CI = 0.06 to 0.17; Fig. 1*A*): Republicans were more likely to judge a news headline as true, resulting in them having slightly higher accuracy for true news (than false news), whereas Democrats had slightly higher accuracy for false news (than true news; Fig. 1*F*). *SI Appendix*, Fig. S3 *C* and *D* shows these effects per study. In sum, Republican, compared to Democrat participants, showed reduced discrimination ability and a slightly more pronounced true-news bias (e.g., naïvety).

#### 1.2.6. Analytical thinking.

Analytical thinking (represented by CRT score) had a strong, credible, and positive effect on discrimination ability (β = 0.66, CI = 0.52 to 0.81; Fig. 1*A*), leading to higher overall accuracy for individuals with higher analytical thinking skills (Fig. 1*G*). Analytical thinking was negatively associated with response bias (β = −0.19, CI = −0.26 to −0.12; Fig. 1*A*), meaning that individuals with higher analytical thinking skills were more inclined to judge a news headline as false, which resulted in greater accuracy for false news (Fig. 1*G*). *SI Appendix*, Fig. S4 *A* and *B* show these effects per study. In sum, higher analytical thinking skills were linked to enhanced discrimination ability and a false-news bias (e.g., caution).

#### 1.2.7. Ideological congruency.

Ideological congruency is operationalized as the alignment of the content of news headlines (i.e., content strongly favoring Democratic views to those strongly favoring Republican views) with participants’ political identity (Democrat, Republican), ranging from strongly incongruent to strongly congruent. We found no credible effect of ideological congruency on discrimination ability (β = 0.1, CI = −0.01 to 0.2; Fig. 1*A*). We did find a strong, credible, and positive effect of ideological congruency on response bias (β = 0.29, CI = 0.22 to 0.37; Fig. 1*A*), showing that participants were more inclined to judge news headline as true (false) if they aligned (misaligned) with their ideological stance. This led to higher accuracy for congruent true headlines than for incongruent true headlines, and the reverse for false headlines: higher accuracy for incongruent false headlines than for congruent false headlines (Fig. 1*H*). *SI Appendix*, Fig. S4 *C* and *D* show these effects per study. In sum, ideological congruency was associated with an increased tendency to believe news headlines (partisan bias) but had no effect on discrimination ability. We also explored the effect of ideological congruency separated by political leaning and found no credible differences (*SI Appendix*, Fig. S5).

#### 1.2.7. Interaction between analytical skills and ideological congruency.

We found no credible effect of the interaction between analytical skills and ideological congruency on discrimination ability (β = 0.04, CI = −0.06 to 0.14; Fig. 1*A*). As for motivated reflection, which is the interaction between analytical thinking and ideological congruency on the response bias, we found a credible and positive effect (β = 0.16, CI = 0.09 to 0.24; Fig. 1*A*). That is, the effect of ideological congruency on response bias was stronger for those with higher analytical skills (Fig. 1*I*). *SI Appendix*, Fig. S6 *A* and *B* show these effects per study.

#### 1.2.8. Familiarity.

Only six studies (from five articles) included a measure of self-reported familiarity with news headlines. Given the extent of missing data, we excluded familiarity from the main SDT model and instead ran a separate complete-case SDT model (*N*_participants_ = 2,619; *N*_choices_ = 50,701; *M*_age_ = 42.13 y, *SD* = 16.23, *range* = 18 to 88). Familiarity did not have an effect on discrimination ability (β = 0.16, CI = −0.03 to 0.3; *SI Appendix*, Fig. S7*A*). However, it had a strong and positive effect on response bias (β = 1.03, CI = 0.67 to 1.34; *SI Appendix*, Fig. S7*A*). Participants were much more likely to label news headlines as true if they were familiar, leading to higher accuracy for familiar true headlines and lower accuracy for familiar false headlines (Fig. 1*J*). In sum, familiarity led to a strong true-news bias. Full regression results for familiarity can be found in *SI Appendix*, Fig. S7 *A* and *B* shows these effects per study.

### 1.3. Additional Analyses: Headline Topic, Platform, and Displayed Source.

**Fig. 2.**
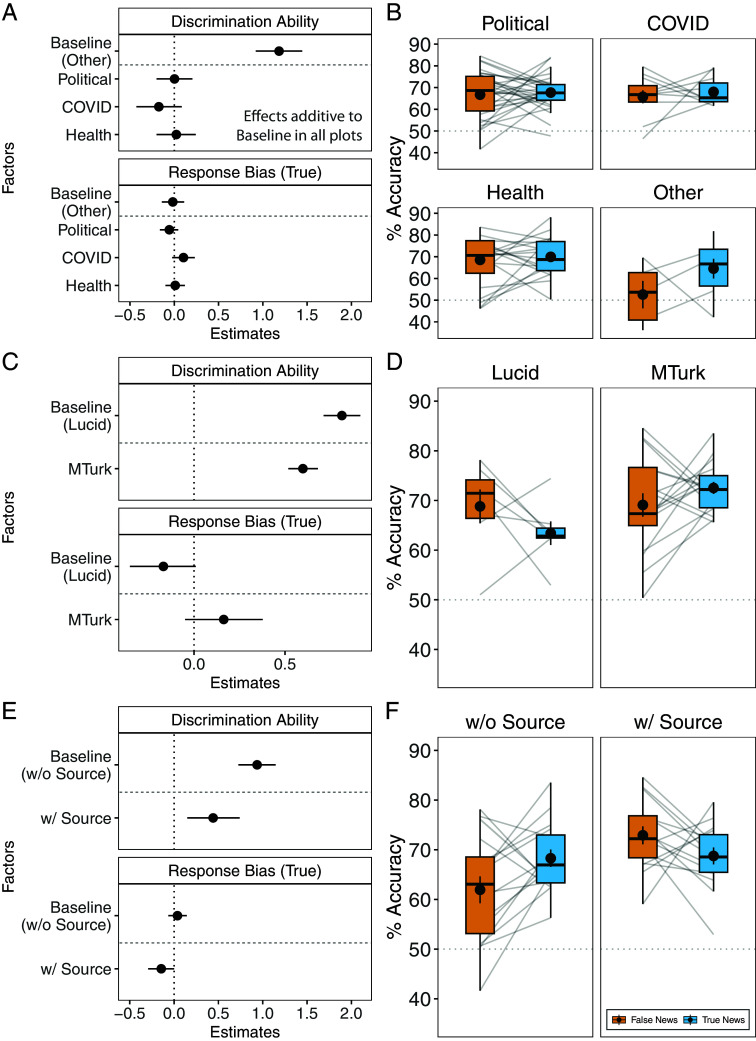
Additional analyses for headline topic (*A*, *B*), study platform (*C*, *D*), and source display (*E*, *F*). (*A*, *C*, and *E*) SDT model estimates for additional analyses. The upper half of each panel shows estimates for discrimination ability, with more positive (negative) values indicating higher (lower) discrimination ability. The lower half shows the estimates for response bias, with more positive (negative) values indicating a higher likelihood to judge headlines as true (false). Dots represent the mean; error bars represent the 95% CI of the posterior distribution. The reference level for each model is presented in brackets after “Baseline”: Baseline (Other): Estimate of discrimination ability and response bias when the headline topic is not related to politics, COVID-19, or general health. (*B*, *D*, and *F*) Accuracy for true and false news headlines for the additional analyses. Lines connect mean true and false news accuracy scores within a study (e.g., across political headlines). Boxplots show the median and the IQR; whiskers indicate an additional 1.5 IQR. Large dots represent the aggregate mean with SE. w/o = without. w = with.

We also analyzed the topic of the headlines (i.e., related to politics, COVID-19, or general health), the crowdsourcing platform (i.e., Lucid or MTurk), and whether the source was displayed (for detailed information on the analyses, see *Materials and Methods* and Fig. 2). Noteworthy results include that the headline topic did not have an effect on discrimination ability, suggesting that the results for discrimination ability are consistent across topic types (Fig. 2 *A* and *B*). Data collection via MTurk, as compared to Lucid, was associated with a strong, positive, and credible effect on discrimination ability (β = 0.6, CI = 0.52 to 0.68; Fig. 2*C*). This led to MTurk participants having greater overall accuracy (Fig. 2*D*). Finally, displaying the headline’s source had a strong, positive, and credible impact on discrimination ability (β = 0.44, CI = 0.15 to 0.74; Fig. 2*E*), resulting in higher overall accuracy when a source was displayed with the headline (Fig. 2*F*). Investigating this further, we found that this effect was stronger for Republican- than Democrat-identifying participants (β = 0.17, CI = 0.09 to 0.26; *SI Appendix*, Fig. S8), suggesting that they benefit more from a source being displayed.

## 2. Discussion

Using 256,337 unique choices made by 11,561 participants across 31 studies, we investigated how four demographic factors—age, gender, education, and political identity—and four psychological factors—analytical thinking, ideological congruency, motivated reflection, and familiarity—impact online misinformation veracity judgments. We additionally analyzed the topic of the headlines (i.e., related to politics, COVID-19, or general health), the platform (i.e., Lucid or MTurk), and whether the source was displayed. We used signal detection theory (SDT; 10) to analyze how these factors are associated with discrimination ability, the ability to distinguish between true and false news, and response bias, the tendency to classify news as true (true-news bias) or false (false-news bias).

**Fig. 3.**
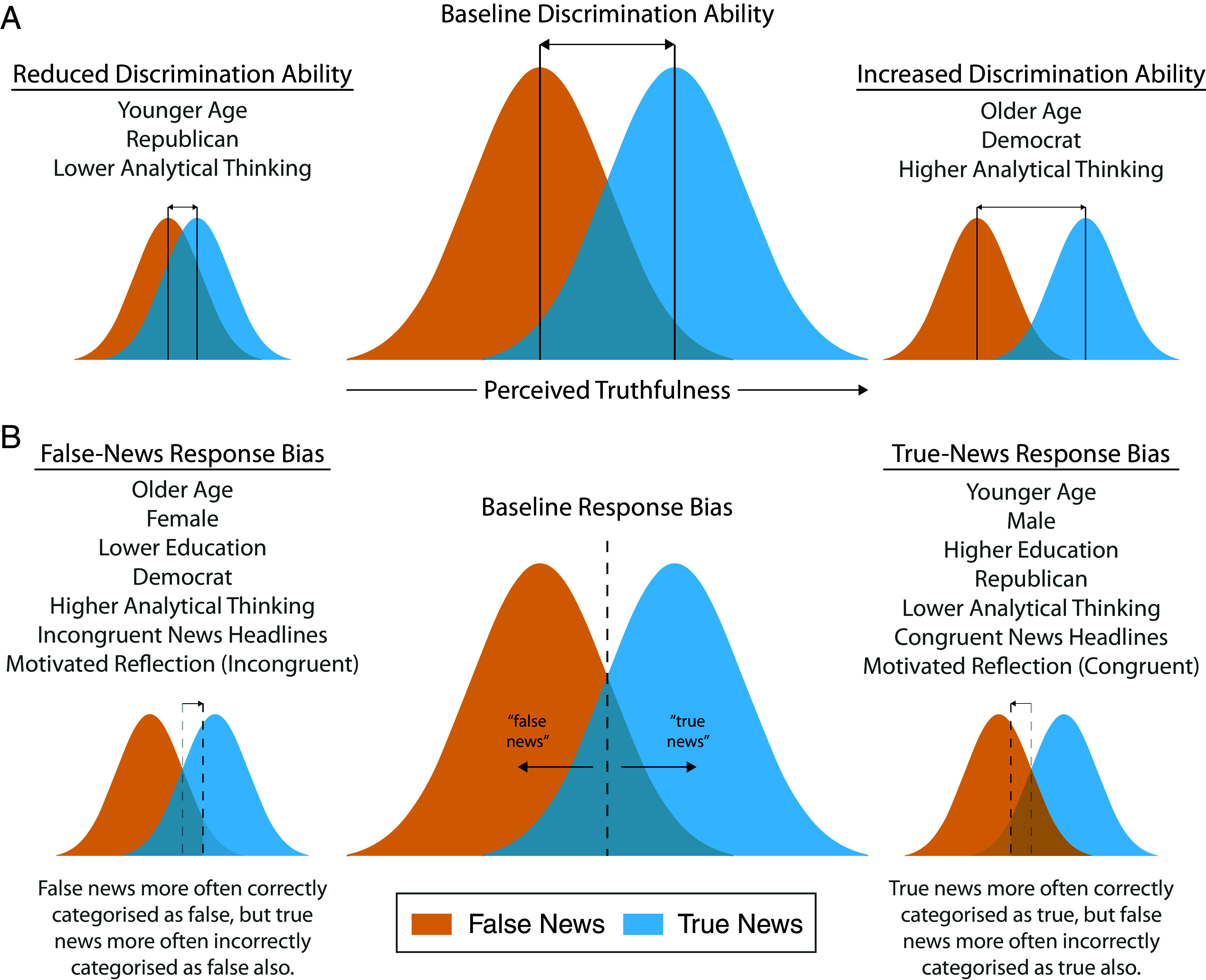
Simplified visual summary of the main signal detection analysis. (*A*) The *Middle* shows a visual representation of baseline discrimination ability. The perceived truthfulness of a news headline is represented by an axis ranging from low truth to high truth, as represented via the two Gaussian distributions. The more the distributions overlap, the more similar the true and false news headlines are perceived (i.e., lower the discrimination ability), whereas the less they overlap, the more dissimilar the true and false headlines are perceived (i.e., higher the discrimination ability). The *Left* shows which factors were associated with reduced discrimination ability and *Right* shows which factors were associated with increased discrimination ability. (*B*) The *Middle* shows baseline response bias, which is determined by a decision criterion (i.e., vertical dashed line). The response for whether a news headline is true or false is dependent on where the headline falls relative to the criterion. If the criterion is placed higher up the perceived truthfulness dimension (*Left*), more evidence is required to treat a news headline as true, hence a headline is treated as true less often, resulting in a false-news response bias. The opposite holds for a true-news response bias (i.e., less evidence is required to render a news headline as true; *Right*). The baseline response bias was neutral in our study. *Left* shows which factors were associated with a false-news response bias and the *Right* shows which factors were associated with a true-news response bias.

Older age and higher analytical thinking skills were associated with better discrimination ability, whereas, identifying as Republican (as opposed to Democrat) was associated with worse discrimination ability. Older age, identifying as female (as opposed to male), and higher analytical thinking skills were associated with a false-news response bias (caution), whereas higher education, identifying as Republican (as opposed to Democrat), ideological congruency, motivated reflection, and familiarity were associated with a true-news response bias (naïvety; Fig. 3). For the additional analyses, headline topic was not associated with discrimination ability, whereas data collection via MTurk (as opposed to Lucid), and displaying the source of news headlines was associated with greater discrimination ability.

While older adults are often considered to be more digitally naïve (13, 14), our findings show that this does not thwart their ability, possibly built up and fostered in the offline world, to accurately discern between true and false news (see also refs. 18, 19, 20). Older adults were also more likely to classify news headlines as false, which can be interpreted as a cautious approach. By contrast, younger adults were less adept at discerning true and false news, and were more naïve, suggesting that their higher digital literacy does not seem to boost their ability to judge online news. These findings raise alarms that younger adults may be more vulnerable, not more resistant, to misinformation. Given these robust results (see also *SI Appendix*, Fig. S2*A*), it is surprising that the effect of age on news veracity judgments has received relatively little attention and lacks a theoretical framework (cf. ref. 21).

Our results raise questions about which factors lead to older adults’ higher discrimination ability. Is this the result of accumulated knowledge over time (e.g., crystallized intelligence; 54), enhanced vocabulary (e.g., enriched semantic memory; 55), or are there more tangible factors like news interest, news consumption patterns, and strategies and heuristics (e.g., source cues) at work that could be harnessed for future interventions? Insights into these questions will also help clarify the paradox between older adults’ veracity judgments and sharing judgments: Despite their higher discrimination ability, what distracts adults—particularly older adults—from news veracity enough for them to end up sharing (the most) false news (for a detailed discussion, see ref. 19)? As a cautionary note, it is important to consider that older adults that take part in online studies may not be representative of their age group, as they likely have higher digital literacy, which could influence the results.

Higher formal education levels were not associated with higher discrimination ability. Surprisingly, we found that more educated individuals had a slight tendency to judge news headlines as true (naïvety). This is striking, especially as education is associated with the development of critical thinking skills, including the ability to challenge assumptions, scrutinize sources, and weigh the pros and cons of information (although see 28). These results, in combination with our findings on age, raise concerns about the adequacy of current educational frameworks, especially as other studies show that highly educated individuals are easily manipulated online by objective-sounding language and official-looking logos and domains (29, 30). Interventions such as lateral reading (30, 56), media literacy (57), and inoculation (prebunking; 58), which are known to improve news veracity judgments and increase caution, may boost such competences once included into educational curricula.

Relatedly, higher analytical thinking skills were robustly associated with better discrimination ability and a tendency to categorize news as false (e.g., caution). This held across partisan lines, echoing previous findings, and cementing the important role of deliberation in enhancing news veracity judgments (4, 39, 40). Despite the null effect of critical thinking skills as learned via education, there is an opportunity here to harness analytical thinking skills to boost (59) news veracity judgments. While thoughtful and deliberative processing of information has been shown to improve veracity judgments in the past (33), it is unclear what exactly the CRT is measuring (60) and what mechanisms are activated during “deliberation”: Are participants putting in more effort (e.g., time), overriding various biases (e.g., truth bias), using more discerning heuristics, or managing to retrieve cues with higher validity (e.g., source credibility) to arrive at correct judgments? If interventions are to more effectively improve veracity judgments, these mechanisms need to be better understood and nurtured.

We found that Democrat participants had a substantially higher discrimination ability compared to Republican participants. This discrepancy represents a growing trend in the literature (33, 34, 35), pointing to the considerable role of political identity in shaping people’s perceptions of truth. One potential explanation lies in the notion that different political groups inhabit distinct informational worlds, both offline and online—a situation that is likely exacerbated by echo chambers, filter bubbles, and microtargeting (31, 32, 61). For instance, studies show that Republican politicians are making fewer evidence-based statements and more belief-based statements (62); that misinformation tends to favor more Republican (conservative) positions, whereas true news tends to favor more Democratic (liberal) positions, thereby skewing perceptions of truth (18, 35); and that Republicans are also more exposed to—and share more—articles from unreliable websites (15, 16, 17).

It is, nonetheless, important to emphasize that the main issue at hand is not whether Democrats or Republicans are better at identifying misinformation but that political identity can shape what people perceive to be true, which can in turn shape their behavior. Because a functioning democracy is ultimately dependent on a shared reality (e.g., that a specific election outcome was legitimate and fair), further work is needed to understand and bridge divergent perceptions of truth. Research could delve deeper into the consequences of exposure to different base rates of partisan news and how that impacts veracity judgments and sharing judgments. Interventions could also aim to emphasize commonalities, shared civic values, and promote respectful dialog across political boundaries to navigate these distinct realities (63).

The implications of how political identity can shape perceptions of truth also become much more apparent in light of ingroup vs. outgroup favoritism. Ideological congruency resulted in a strong true-news bias (see also refs. 4 and 39), both for Democrat and Repubilcan participants. That is, news that was congruent (incongruent) with one’s political identity was seen as true (false) more often. One explanation for this lies with the identity-based model of political belief (5, 64), positing that people need to maintain a positive view of their ingroup (political party) in relation to their outgroup. This has led some to argue that partisan differences in vaccinations and public-health behaviors can be explained by such identity-driven motivations (65). Note that congruent news may also be more familiar news, resulting in the congruency effect partially, or fully, being a familiarity effect in disguise. This, in turn, could attenuate the role that identity-driven motivations play in the susceptibility to misinformation. How these two drivers relate deserves more research attention. Overall, as ideological congruency predominantly affects response bias, effective interventions should encourage individuals to objectively evaluate information, irrespective of its ideological alignment. We also encourage future research to investigate the cues that people treat as signals for ideological congruency in order to disentangle the mechanisms behind ideological congruency (e.g., see refs. 5 and 64).

Individuals with higher analytical thinking skills are known to exhibit motivated reasoning. It is hypothesized that they use their cognitive abilities to selectively process information to align with their preexisting beliefs (6, 42, 66). However, this is a heavily contended concept, with numerous studies challenging its reliability (33, 43, 44, 45). It has been argued that underpowered studies might be at the root of these mixed findings (66). In response, studies using large and representative samples have failed to replicate the effect (43, 44). Our analysis reveals a robust, albeit small, effect of motivated reflection (*SI Appendix*, Fig. S6). Such a small effect could explain why past studies have failed to replicate this effect, but this does not explain why the higher powered studies have failed to find it. We believe the mixed findings may partly stem from varying definitions of motivated reasoning, both semantically and methodologically. In some instances, it is referred to as motivated reasoning (20, 42), others refer to it as motivated numeracy (6), and motivated reflection (the present study; 39). This inconsistent terminology is also associated with varying operationalizations of “cognitive ability” in motivated reasoning (e.g., numeracy, literacy, education, intelligence). To clarify this ambiguity, a thorough examination of studies on motivated reasoning (numeracy/reflection) is needed, focusing on specific operational definitions to determine whether we are comparing equivalent or disparate phenomena. In the present case, we recommend treating the effect of motivated reflection with caution due to its modest size.

Finally, there was a strong association between familiarity with a news headline and the likelihood that participants would believe it to be true, regardless of whether it was or not (39, 40). Familiarity can easily be gamed online, where similar headlines can appear and reappear in endless news feeds, especially within echo chambers. Generally, it may be beneficial to trust familiar information: Doing so can reduce cognitive load and simplify decision-making processes. However, this adaptive process becomes maladaptive in an environment where familiarity can be manipulated—at an unprecedented speed and sophistication—and is not in itself indicative of veracity. Unfortunately, interventions to eliminate the effect of familiarity have largely been unsuccessful. Though, interventions that ask participants to assess the familiarity or truthfulness of news headlines have shown some promise in reducing the effect (49). As the familiarity effect has been studied in other domains, such as with trivia statements (67), this literature could be reviewed to identify interventions that have successfully reduced the effect. Note, however, that our findings on familiarity are based on only six studies due to our eligibility criteria, which excluded studies that experimentally manipulated prior exposure to news headlines. Given the robustness of the familiarity effect in prior studies, we are nonetheless confident in these results (for recent reviews, see refs. 46 and 49).

In our additional analyses, we found that the results for discrimination ability hold across different types of news headline topics (i.e., related to politics, COVID-19, or general health). These results generally speak to the robustness of our findings across different headline topics. We also found that MTurk participants exhibited higher discrimination ability than Lucid users, highlighting that veracity judgments can be systematically biased depending on the recruitment platform. This could be due to various reasons, including task engagement of participants (e.g., quickly clicking through), experience with news related tasks, and how the quality of participants is maintained on the platforms. Conducting studies across multiple platforms—where possible—may mitigate this effect.

People were better at judging the veracity of a headline if a source was also displayed alongside the news headline. Studies looking into source credibility (e.g., that manipulate the size of sources or the partisan alignment of sources) have found mixed results (68, 69). Our results, however, clearly suggest that the presence of a source is used as a cue for veracity. Naturally, relying on source credibility as a heuristic is adaptive, especially given established trustworthiness of sources. This, however, can easily be gamed in the age of artificial intelligence, reinforcing the need to build and maintain trustworthy institutions and to maintain trust in these institutions. Beyond this, at a methodological level, omitting source display can lead to issues that conflate source credibility with content veracity (e.g., isolating the direct effects of content characteristics on judgment veracity). It can also, for example, make it challenging to assess efficacy uniformly across intervention studies that vary in their display of source, or studies that mix source display altogether.

We find no evidence for an overall response bias across studies. However, there was substantial variation in response bias across studies, with studies exhibiting a true-news bias, a false-news bias, or no bias (*SI Appendix*, Fig. S1*B*). This variability may be indicative of broader study-level features, such as news headline selection. To illustrate, while false news headlines are primarily sourced from fact-checking organizations (e.g., Snopes), true news headlines are largely derived from mainstream sources and may exhibit systematic differences. The reverse scenario may also hold, where fact-checked headlines differ systematically from true-news headlines. Additionally, participants’ responses may be influenced by experimental and contextual demands (e.g., true-false base rates, participants’ awareness of misinformation in the study). To probe the influence of experiment demands, studies could employ experience-sampling methods or social media simulators (70, 71).

Several limitations need to be considered. First, the included news headlines do not fully represent the spectrum of news encountered online. Second, almost all studies use an equal distribution of true and false news, which does not reflect base rates encountered online (72, 73). Third, not all studies collected data on every demographic and psychological factor, leading to some data being imputed (*Materials and Methods*). Finally, our analysis focused on a US-only sample and simplified gender and political identity into binary categories, which does not capture their full complexity. In the case of political identity, this also meant excluding Independents, who are a sizable US minority (74).

Given the multitude of demographic and psychological factors shaping misinformation veracity judgments, research should adopt a multifaceted and multipronged approach (75, 76). The insights provided by our meta-analysis establish the robustness (or lack thereof) of demographic and psychological factors, touch upon key debates in the literature, pave the way for future research (Table 1), and highlight the need for tailored interventions that take these factors into account. Beyond this, we call for more representative and ecologically valid research methodologies to enhance the generalizability and robustness of the findings in our fast-paced digital world. Citizens’ ability to withstand the onslaught of misinformation will likely be key to successfully managing global policy challenges ranging from climate change, violent conflicts, pandemic preparedness, and democratic backsliding.

**Table 1.** Key recommendations for future research

**Include relevant variables.** Important variables like age and political identity are often collected but not adequately analyzed. Future research could systematically include and measure these variables to avoid omitting meaningful variance.
**Go beyond overall accuracy.** Using analytical frameworks like SDT that can assess discrimination ability and response bias simultaneously is crucial. This approach provides a more nuanced understanding of how people discern truth in news that can be used to develop more targeted interventions.
**Incorporate reaction time data.** Including reaction time data in future studies could offer deeper insights into the cognitive mechanisms behind news veracity judgments (e.g., via a drift–diffusion modeling approach; 77, 78).
**Select diverse headlines.** The number and types of headlines used in studies should be carefully considered. A representative sample of headlines, reflective of the broader online ecosystem, is essential for more accurate insights. More ecologically valid studies are critical for generalizability.
**Understand veracity as part of a larger puzzle.** Veracity is just one aspect of misinformation. A view that considers other elements of news consumption and sharing is necessary.
**Dive deeper into headline characteristics.** The headlines used in studies are complex stimuli. A deeper analysis of headline characteristics (e.g., text complexity, emotion) could help shed light on veracity judgments and sharing judgments.
**Expand beyond headlines.** While our focus has been on news headlines, there is a larger context of news consumption that should be explored, including other forms of news media consumption (e.g., full-length articles; offline consumption, instant messaging platforms).
**Practice Open Science.** Most of the necessary data for our meta-analysis were accessible. Researchers should maintain transparency, making data readily available for replication and further analysis.
**Improve data coding and documentation.** Improvements in how data are coded and documented would greatly aid future research. Providing a detailed codebook, for instance, would facilitate data comprehension and reusability.
**Move beyond WEIRD samples (79).** This study focused on US-based sample populations, as much of the current research is conducted there. We encourage research that covers other geographical areas, in particular countries that are not based on a two-party political system.

## Materials and Methods

This meta-analysis was preregistered and conducted in line with the Preferred Reporting Items for Systematic Reviews and Meta-Analysis guidelines (PRISMA; 80; *SI Appendix*, Fig. S9). The preregistration, along with all the materials to reproduce our analyses, are available on OSF (https://osf.io/s2ejg/). Deviations from the preregistration can be found in “Protocol Deviations” on OSF (https://osf.io/s2ejg/). Our study did not require ethical approval as we reanalyzed preexisting data. Data that were not publicly available were requested from the authors.

### Eligibility Criteria.

Table 2 presents the study eligibility criteria.

**Table 2.** Eligibility criteria

Criteria	Description
**Participants**	Only healthy (i.e., nonclinical) adults who were 18 y of age or older and based in the United States were included.
**Study design**	Participants judged the veracity of a series of news headlines. Headlines (e.g., the Misinformation Susceptibility Test; 86) could be accompanied by an image, a byline, and/or a source. Studies all included both true and false news headlines that were viewed individually (e.g., no infinite scroll). Studies that provided immediate feedback were excluded.
**Outcome measure**	Eligible veracity question framing included whether the news headline was accurate, real, fake, trustworthy, believable, credible, or manipulative. Acceptable responses were either binary (true/false) or derived from even-numbered Likert scales (odd-numbered Likert scales cannot be binarized, as the middle point cannot be assigned to either side of the scale). Note that previous research has shown little difference in veracity judgments across different question framings and response formats (37).
**Control data**	Data from control treatments in studies featuring both an intervention and a control were included if they satisfied all other inclusion criteria. Data were excluded if participants had to assess the news headlines on aspects other than its veracity (e.g., sharing intentions). Exceptions were made for evaluations of news headlines’ familiarity and participants’ confidence in their veracity judgments. Familiarity was considered because we were interested in its effect. Since confidence levels can be viewed as meta-judgments to veracity judgments, we determined that this aspect would not alter participants’ responses (see also ref. 37).
**Model input**	Data that could not be converted to the desired model input were excluded (*Statistical Analysis*). For example, data were excluded if the political identity of participants was measured using an odd-numbered Likert scale, which cannot be binarized.
**Time frame**	Studies where participants were asked to rerate headlines they had already been shown were excluded (i.e., one-time ratings of news headlines only).
**Study year**	Studies conducted before 2004, the year Facebook was founded, were excluded due to our interest in news headlines as seen on social media (i.e., image and headline).
**Language**	Only studies that used English-language headlines were considered.
**Publication status**	Published and unpublished (e.g., preprints; shelved data) original research was considered, excluding reviews and meta-analyses.
**Data availability**	Studies were excluded if their raw data were not accessible online and remained unavailable after multiple unanswered data requests. A list of studies excluded due to inaccessible data can be found in Search Results on OSF (https://osf.io/s2ejg/).

### Search Strategy.

We used two general databases, Web of Science and Scopus, and one subject-specific database, PsycINFO, to search for relevant literature. This search strategy was developed with—and tested against—a preselected list of six articles that matched the eligibility criteria (33, 37, 81, 82, 83). The search string was developed to include terms from four main categories of interest: news AND misinformation AND veracity AND human. Exact search terms for Web of Science, Scopus, and PsycINFO are listed in *SI Appendix*, Table S1. The search was limited to English-language articles, conference papers, and early access papers from 2004 until November 2022. To identify unpublished and shelved data, we also searched PsyArXiv Preprints and sent email requests to the Society for Judgment and Decision Making and the Fake News Sci mailing list. For a detailed breakdown of the search strategy, including exact search terms for each platform and dates, see “Search Syntax” on OSF (https://osf.io/s2ejg/).

### Screening.

We selected 21 articles (encompassing 31 studies) for data extraction (for an overview, see *SI Appendix*, Fig. S9). EndNote (https://endnote.com/) was used to track and manage search results (*N* = 4,666), including deduplication. The lead author (MS) first conducted a title-only screening of articles (*N* = 3,314) in order to exclude articles that were clearly irrelevant (e.g., articles that used machine learning to detect misinformation, non-US-based samples; *N*_remaining_ = 1,261). Next, two coders (MS and NE) screened 100 titles and abstracts against the eligibility criteria to clarify questions and assess interrater agreement. This served as a training round to ensure that both raters’ subjective ratings agreed with the eligibility criteria, with disagreements overcome through discussion. We had preregistered to repeat this step if the interrater agreement was below 90%. In our case, an interrater agreement of 87% was reached in each of two consecutive attempts (*n* = 200), so we decided to proceed to the next step: We used Abstrackr (84) to make title- and abstract-based decisions with reference to the eligibility criteria (*N*_total_ = 1,257). Article titles and abstracts (along with other fields, e.g., journal and authors) were displayed on Abstrackr. The articles were randomized for both MS and NE. MS and NE then screened the full texts of all remaining articles for eligibility (*N* = 267); when uncertain, they consulted RHJMK and ANT. An interrater agreement of 87% and an interrater reliability of 0.64 (Cohen’s *Kappa*, *P*< 0.001; using the irr R package; 85) was reached. Four further articles were identified via mailing lists but did not meet the inclusion criteria. *SI Appendix*, Table S2 presents the final list of articles, including the number of studies per article, each study’s sample size, and number of headlines. See also “Search Results” on OSF (https://osf.io/s2ejg/) with the full—and final—lists of studies, including notes on disagreements.

### Data Extraction.

Data from all studies were recorded and extracted by MS and NE. The data comprise five categories: study and participants, news headlines (e.g., veracity, political leaning), demographic factors, psychological factors, and veracity judgments. *SI Appendix*, Table S3 outlines each extracted variable. *SI Appendix*, Table S4 provides basic descriptive statistics for the sample, including age, gender, education, and political identity. Further descriptive statistics for age (*SI Appendix*, Fig. S10), gender and political identity (*SI Appendix*, Fig. S11), education (*SI Appendix*, Fig. S12), and analytical thinking (*SI Appendix*, Fig. S13) are also provided. Correlations between the factors are provided in *SI Appendix*, Fig. S14.

#### Preprocessing and missing data.

We had to standardize data across studies due to variation in data collection methods. Notably, this included converting data points from various scale response modes (e.g., a 6-point scale) into a binary format. Because education was measured inconsistently across studies, we converted it into three levels: secondary (including those who did not complete secondary education), undergraduate, and graduate, and turned it into a ridit score (87) to better account for the ordinal nature of the data. Due to the broad scope of this meta-analysis, not all of the 31 studies included the demographic and psychological factors of interest. This resulted in missing data for education (10.71% overall; 4/31 studies), political identity (6.52% overall; 3/31 studies), analytical thinking (31.3% overall; 8/31 studies), and familiarity (80.23% overall; 25/31 studies). These data were mean-imputed. Due to the large amount of missing data for familiarity, however, we excluded it from the main analysis and ran a complete-case analysis instead (see *Complete-Case Familiarity SDT Model*).

To determine ideological congruency, we required the political leaning of a given news headline (i.e., whether it favored Democrats or Republicans), which was missing in 16/31 studies. We therefore used GPT4 to categorize the political leanings of news headlines (88). We first assessed the quality of GPT4 ratings by prompting GPT4 to code the political leanings of headlines we had already categorized; this resulted in high interrater agreement (88%) and interrater reliability of 0.78 (Cohen’s *Kappa*, *P*< 0.001; using the irr R package; 85). Given the high reliability, we used GPT4 to categorize the political leanings of all headlines into the following categories: strongly Republican, moderately Republican, lean Republican, neutral, lean Democratic, moderately Democratic, strongly Democratic (for more on the procedure, see *SI Appendix*, Figs. S15 and S16 and section S1). These ratings were combined with the political leanings of the participants (Democrat, Republican) to create a measure of ideological congruency. For instance, when the political leaning of the participant and the news headline leaning aligned (e.g., both Democratic), this was deemed congruent. Conversely, where there was a mismatch, this was deemed incongruent (for exact coding, see below).

### Statistical Analysis: SDT Using Bayesian Generalized Linear Mixed-Effects Modeling.

#### Main SDT analysis.

We conducted an SDT analysis using a mixed-effects model. We used a Bayesian generalized linear mixed-effects model (GLMM) with the R package brms (89) assuming a Bernoulli-distributed response with a probit link function. This implementation of a mixed-effects signal detection model allowed us to differentiate between discrimination ability and response bias (for a detailed overview, see refs. 90, 91, 92; see also *SI Appendix*, section S2). In the GLMM, we used participants’ response to the veracity question (i.e., veracity_response_binary; false, true) as the outcome variable. The predictor variables were item_veracity (whether the headline is false or true); part_age; part_gender (male, female); part_education (i.e., secondary, undergraduate, and graduate turned into ridit scores); part_political_identity_binary (Democrat, Republican); CRT (proportion of correct responses on the CRT; range 0 to 1); ideological_congruency (the alignment of the political lean of news headlines with participants’ political identity; strongly incongruent, moderately incongruent, lean incongruent, neutral, lean congruent, moderately congruent, strongly congruent); and motivated_reflection (interaction between CRT × ideological_congruency). Finally, for random effects, we accounted for variability at multiple levels: we accounted for individual differences in discrimination ability and response biases among participants by including participant (part_id) as a random intercept and item_veracity as a random slope, and we included study-specific intercept and slope variations by adding study (study_id) as a random intercept and adding all of the fixed effects as random slopes into the model. We also accounted for headline-specific variations by adding news headline (item_id) as random intercepts (for full model specification, see *SI Appendix*, section S2).

The intercept in this regression model reflects the response bias (i.e., the overall likelihood to classify a given headline as true) and the predictors’ coefficients reflect their influence on this response bias. The only exception to this is when the coefficients include item_veracity, which indicates whether the headline is actually true or false. A positive estimate of headline veracity indicates higher ability to identify true news as true and false news as false (i.e., discrimination ability). The influence of the predictors on discrimination ability is, thus, inferred via the estimates of their interactions with headline veracity. To aid with model interpretation, all predictors were mean centered (i.e., value − mean). Part_age, part_education, CRT, and ideological_congruency were also divided by two SDs after mean centering (93). For this model and those detailed below, the parameter estimates were generated by simulating four Markov chain Monte Carlo (MCMC) chains with 10,000 iterations each, discarding the first 5,000 as burn-in. We used the Gelman-Ruben statistic (Rhat) and visually inspected the Markov chains to ensure that all chains had converged. We report the mean of the posterior distribution and the 95% CI.

#### Complete-case familiarity SDT model.

As only six studies (from five articles) included a measurement of familiarity, we ran a separate complete-case SDT model for familiarity (*N*_participants_ = 2,619; *N*_choices_ = 50,701; *M*_age_ = 42.13 y, *SD* = 16.23, *range* = 18 to 88), comprising 51.93% female (48.07% male) and 42.04% Republican (57.96% Democrat). While familiarity was complete-case, we had missing data for education (24%; 1/6 studies) and analytical thinking (36.52%; 2/6 studies). As with previous data, these values were mean imputed. The model was identical to the main model described above, apart from the following exceptions. This model included headline familiarity—familiarity_binary (unfamiliar, familiar)—as an additional predictor variable. This was mean centered. To account for study-specific intercept and slope variations, we used study (study_id) as a random intercept and only familiarity_binary as a random slope in the model. This was done to avoid convergence issues given the limited number of studies in the model.

#### Additional analyses.

We used the same method as in the main SDT analysis to study the impact of headline topic (i.e., related to politics, COVID-19, and general health), the platform (i.e., Lucid or MTurk), and whether the source was displayed on discrimination ability and response bias. See *Main SDT Analysis* above for guidelines on model interpretation.

#### Headline topic.

We used participants’ response to the veracity question (i.e., veracity_response_binary; false, true) as the outcome variable. The predictor variables were item_veracity (whether the headline is false or true), topic_political (whether the news headline was political; 0, 1), topic_covid (whether the headline was COVID-19 related or not; 0, 1), and topic_health (whether the headline was related to general health or not; 0, 1). Finally, for random effects, at the participant level, we modeled random intercepts for participants (part_id) and random slopes for item_veracity; we also modeled study (study_id), and news headlines (item_id) as random intercepts.

We used GPT 4 to generate the topic categories of the headlines. For each headline, the following prompts were used: 1) “Is the headline related to political issues or discussions? Answer with 0 for no and 1 for yes”; 2) “Is the headline related to COVID-19? Answer with 0 for no and 1 for yes”; and 3) “Is the headline related to health or medical information? Answer with 0 for no and 1 for yes.”

#### Platform.

All aspects of the model remained the same apart from the predictor variables, which were item_veracity (whether the headline is false or true) and study_platform (Lucid, MTurk).

#### Source.

All aspects of the model remained the same apart from the predictor variables, which were item_veracity (whether the headline is false or true) and item_source (not present, present).

## Supplementary Material

Appendix 01 (PDF)

## Data Availability

All data have been anonymized and deposited on OSF (https://osf.io/s2ejg/; 94).
