# Supplementary material for: Susceptibility to online misinformation: A systematic meta-analysis of demographic and psychological factors
Source: Proc Natl Acad Sci U S A. 2024 Nov 12;121(47):e2409329121. doi: 10.1073/pnas.2409329121 (PMC11588074; doi:10.1073/pnas.2409329121)
Supplement: Supplementary file 1 — Appendix 01 (PDF) [file pnas.2409329121.sapp.pdf]

PNAS

Supporting Information for

Susceptibility to Online Misinformation: A Systematic Meta-Analysis of Demographic and Psychological Factors.

Mubashir Sultan, Alan N. Tump, Nina Ehmann, Philipp Lorenz-Spreen, Ralph Hertwig, Anton Gollwitzer, and Ralf H. J. M. Kurvers

Mubashir Sultan
Email: mubashirsultan@outlook.com

This PDF file includes:

- Figures S1 to S16
- Tables S1 to S4
- Section S1 to S2

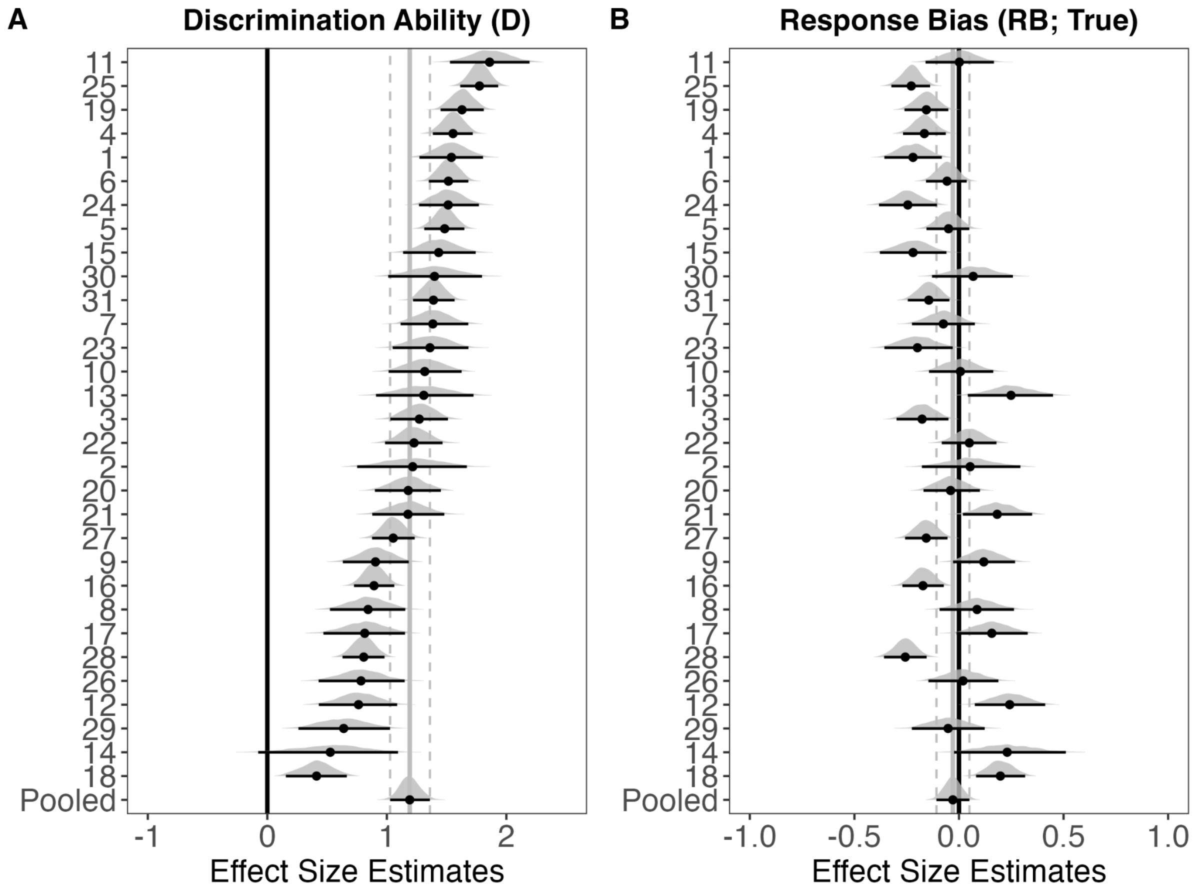

Figure S1. *Study-level SDT model estimates for discrimination ability and response bias. All estimates derive from the SDT model in the main text (Figure 1A). Panel A shows the estimates for discrimination ability, with more positive (negative) values indicating higher (lower) discrimination ability. Panel B shows the estimates for response bias, with more positive (negative) values indicating a higher likelihood to judge headlines as true (false). Pooled: Overall estimate of discrimination ability (A) and response bias (B). Dots represent the mean and the error bars the 95% CI of the posterior distribution. All factors were mean centred. Studies are ordered from highest to lowest discrimination ability, and study numbers correspond to study numbers in Table S2.*

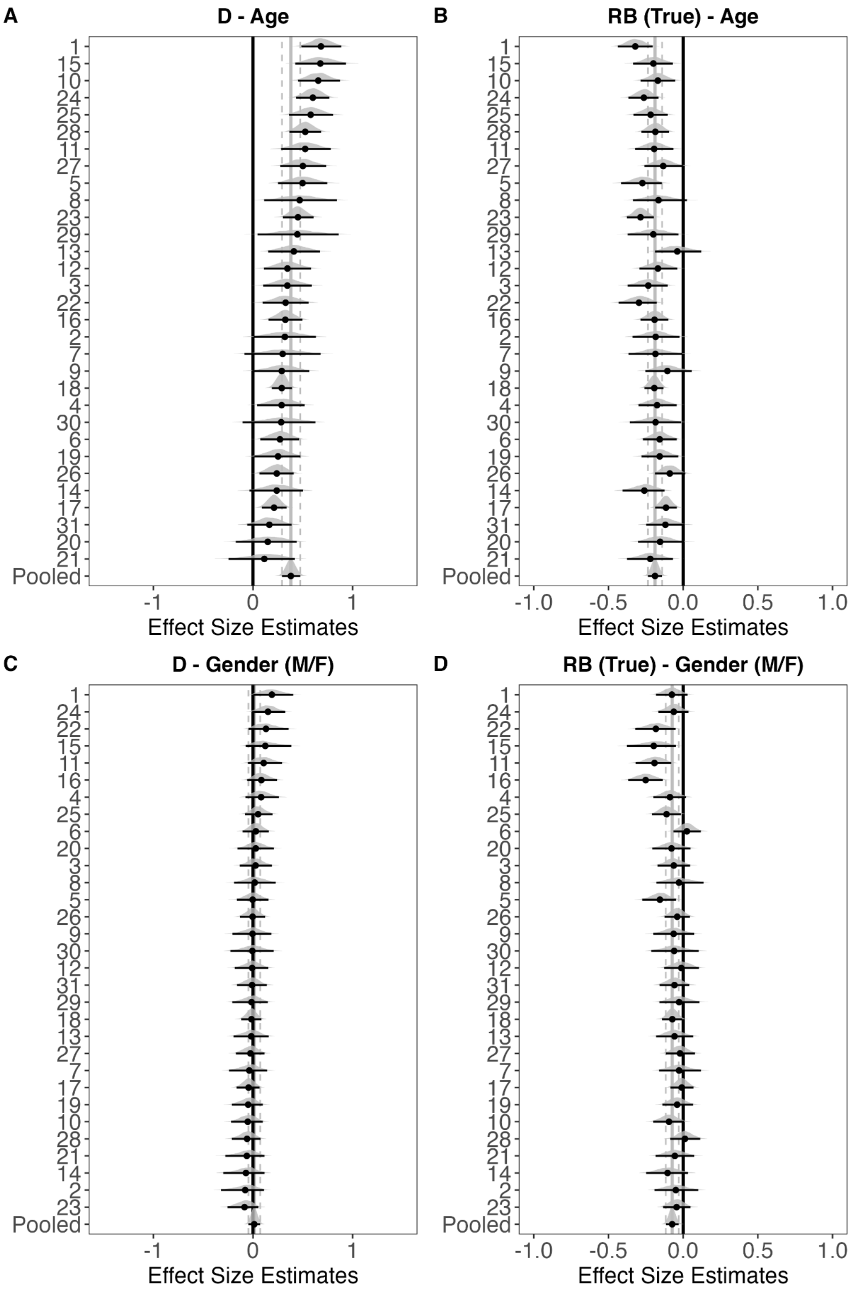

Figure S2. *Study-level SDT estimates for discrimination ability and response bias for age and gender. All estimates derive from the SDT model in the main text (Figure 1A). Left panels (A, C) show the estimates for discrimination ability, with more positive (negative) values indicating higher (lower) discrimination ability. Right panels (B, D) show the estimates for response bias, with more positive (negative) values indicating a higher likelihood to judge headlines as true (false). D = discrimination ability. RB = response bias. Pooled: Overall estimate of discrimination ability (A, C) and response bias (B, D). Dots represent the mean and the error bars the 95% CI of the posterior distribution. All factors were mean centred. Studies in the upper (lower) panels are ordered from highest to lowest effect of age (gender) on discrimination ability, and study numbers correspond to study numbers in Table S2.*

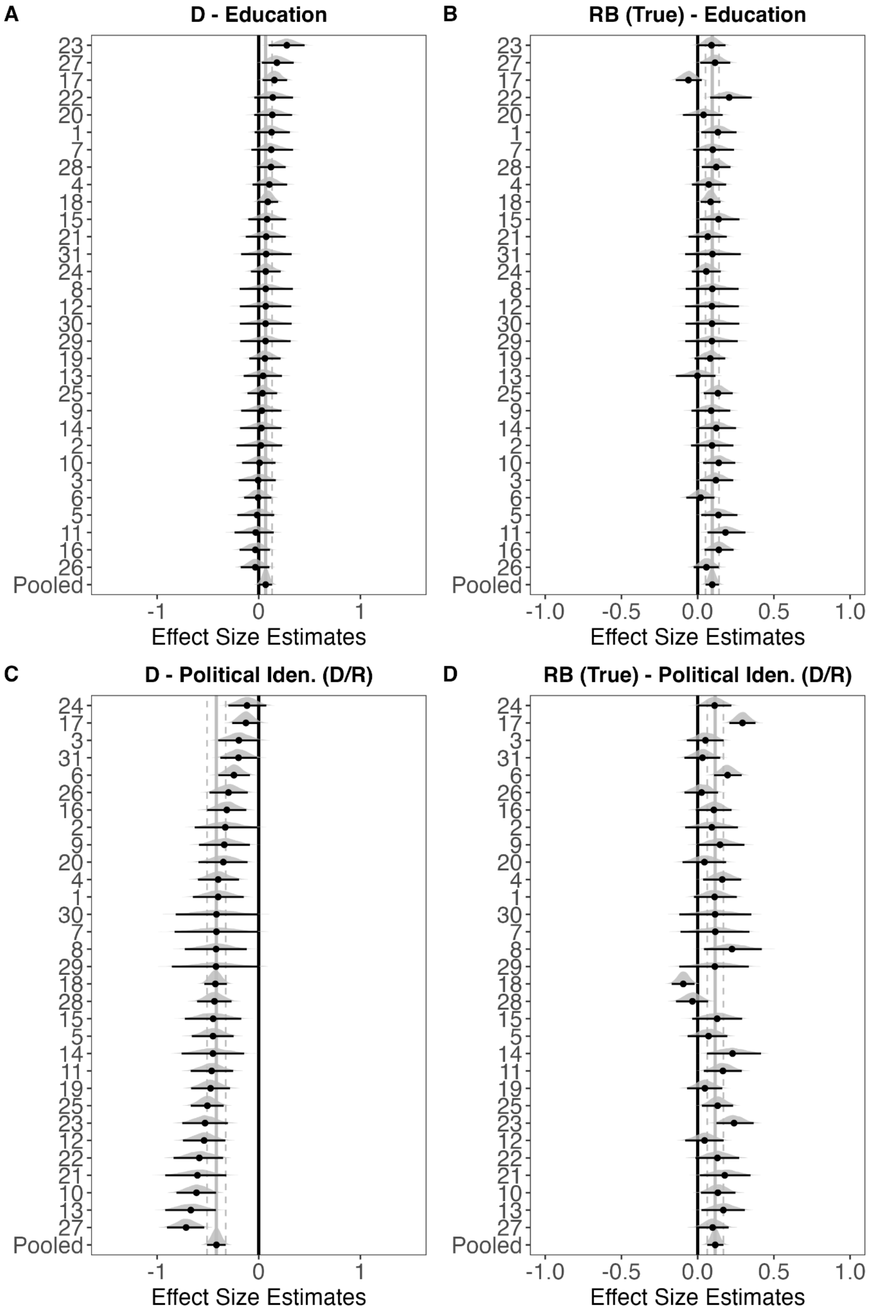

Figure S3. *Study-level SDT estimates for discrimination ability and response bias for education and political identity. All estimates derive from the SDT model in the main text (Figure 1A). Left panels (A, C) show the estimates for discrimination ability, with more positive (negative) values indicating higher (lower) discrimination ability. Right panels (B, D) show the estimates for response bias, with more positive (negative) values indicating a higher likelihood to judge headlines as true (false). D = discrimination ability. RB = response bias. Pooled: Overall estimate of discrimination ability (A, C) and response bias (B, D). Dots represent the mean and the error bars the 95% CI of the posterior distribution. All factors were mean centred. Studies in the upper (lower) panels are ordered from highest to lowest effect of education (political identity) on discrimination ability, and study numbers correspond to study numbers in Table S2.*

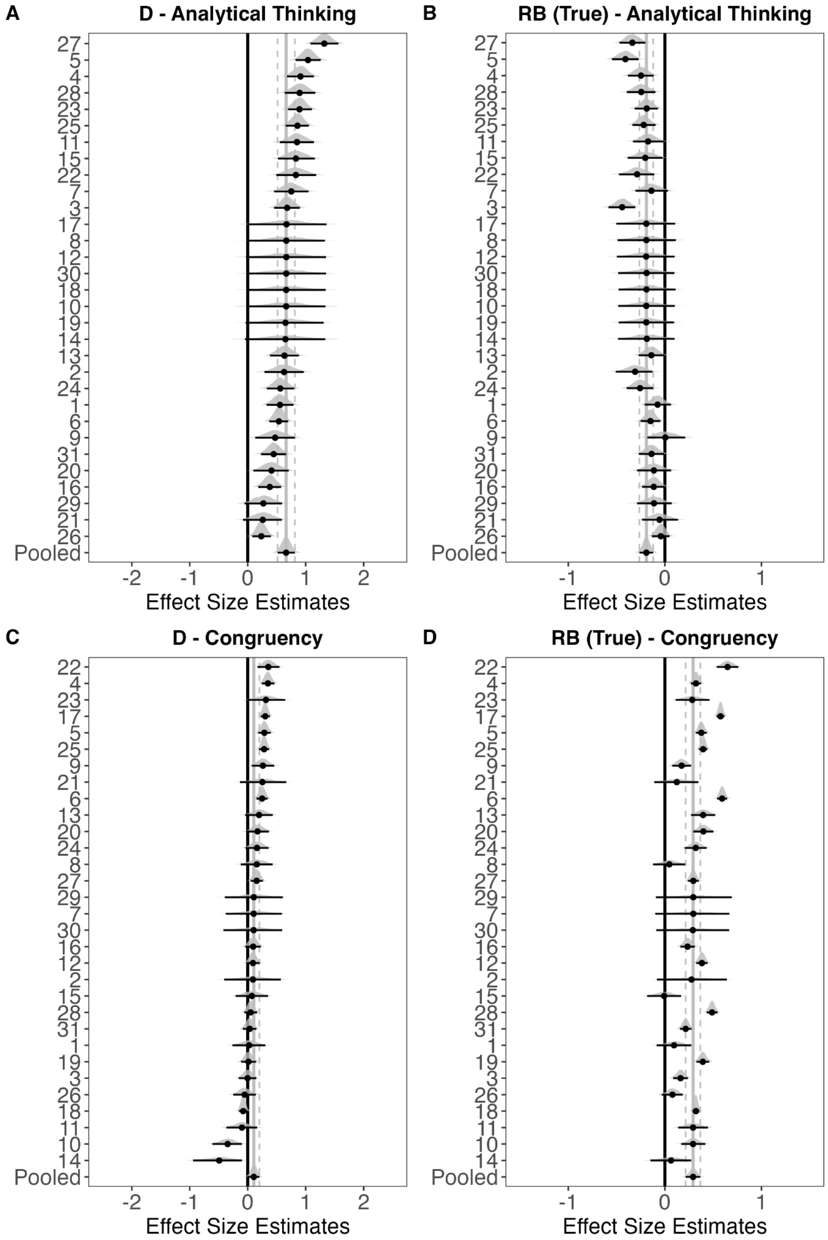

Figure S4. *Study-level SDT estimates for discrimination ability and response bias for analytical thinking and ideological congruency. All estimates derive from the SDT model in the main text (Figure 1A). Left panels (A, C) show the estimates for discrimination ability, with more positive (negative) values indicating higher discrimination ability. Right panels (B, D) show the estimates for response bias, with more positive (negative) values indicating a higher (lower) likelihood to judge headlines as true (false). D = discrimination ability. RB = response bias. Pooled: Overall estimate of discrimination ability (A, C) and response bias (B, D). Dots represent the mean and the error bars the 95% CI of the posterior distribution. All factors were mean centred. Studies in the upper (lower) panels are ordered from highest to lowest effect of analytical thinking (congruency) on discrimination ability, and study numbers correspond to study numbers in Table S2.*

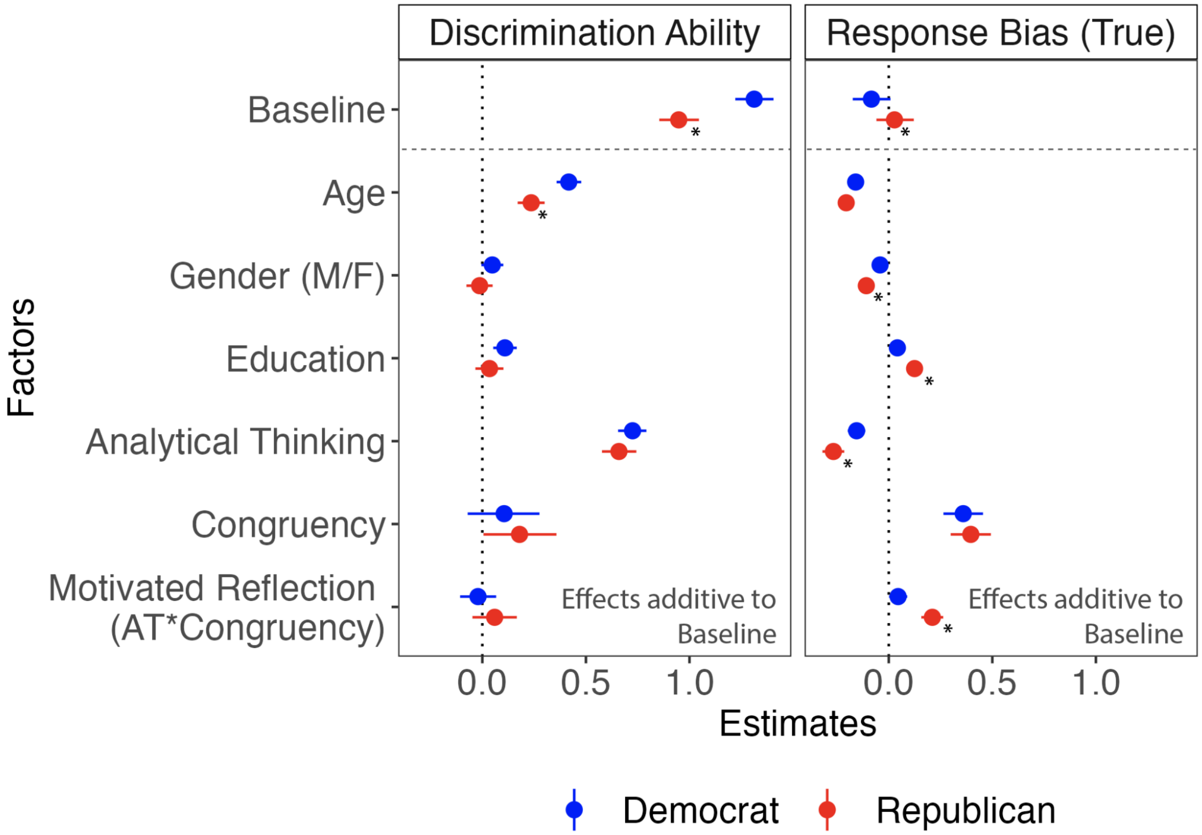

Figure S5. *SDT model estimates separated by Democrat (blue) and Republican (red) participants. The left panel shows the estimates for discrimination ability, with more positive (negative) values indicating higher (lower) discrimination ability. The right panel shows the estimates for response bias, with more positive (negative) values indicating a higher likelihood to judge headlines as true (false). Baseline: Overall estimate of discrimination ability (left panel) and response bias (right panel), separated for Democrats and Republicans. Gender (M/F) = coded Male to Female. Congruency = ideological congruency. AT*Congruency = interaction between Analytical Thinking and Congruency. Dots represent the mean and the error bars the 95% CI of the posterior distribution. Note that all factors were mean centred. * = credibly different effects between Democrat and Republican participants.*

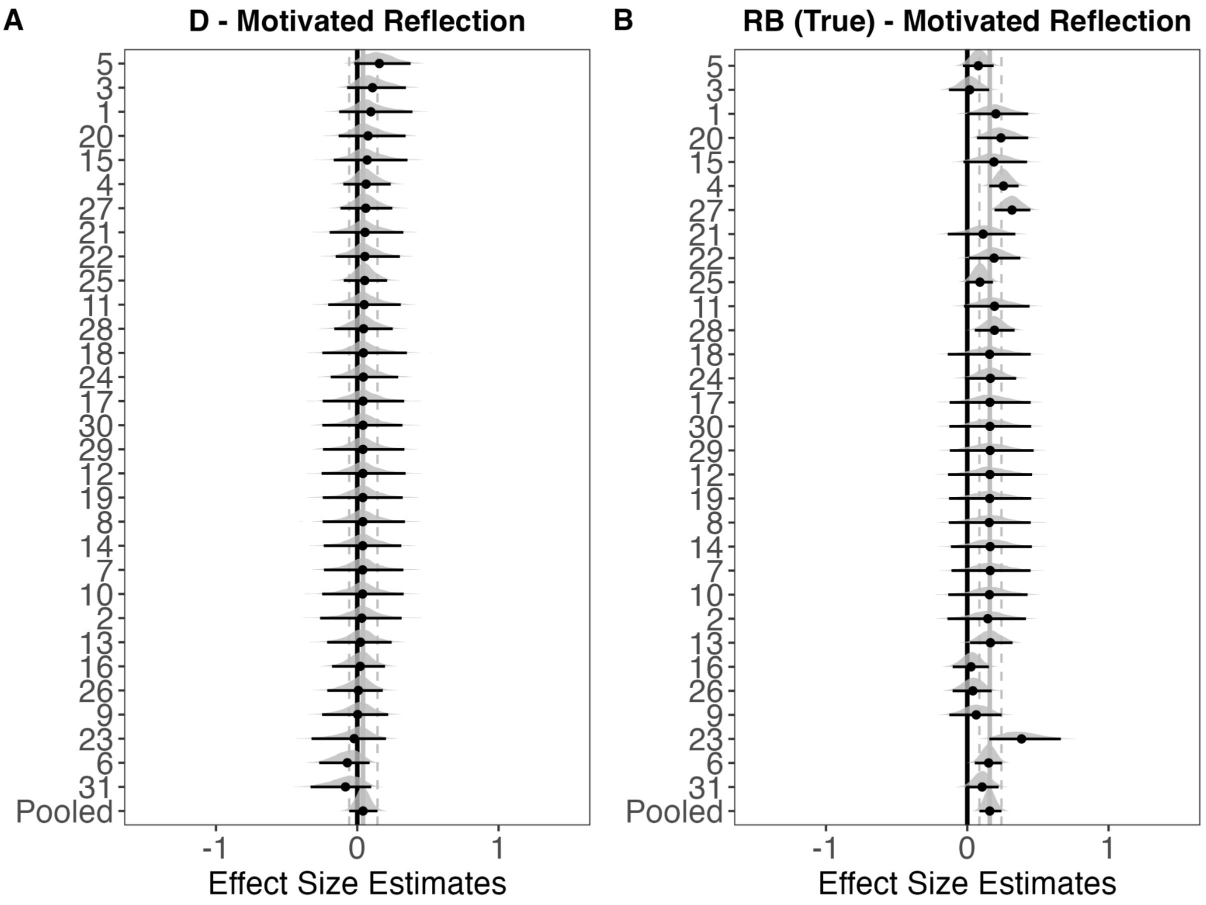

Figure S6. *Study-level SDT estimates for discrimination ability and response bias for motivated reflection. All estimates derive from the SDT model in the main text (Figure 1A). Left panel (A) shows the estimates for discrimination ability, with more positive (negative) values indicating higher (lower) discrimination ability. Right panel (B) shows the estimates for response bias, with more positive (negative) values indicating a higher likelihood to judge headlines as true (false). D = discrimination ability. RB = response bias. Pooled: Overall estimate of discrimination ability (A) and response bias (B). Dots represent the mean and the error bars the 95% CI of the posterior distribution. All factors were mean centred. Studies are ordered from highest to lowest effect of motivation reflection on discrimination ability, and study numbers correspond to study numbers in Table S2.*

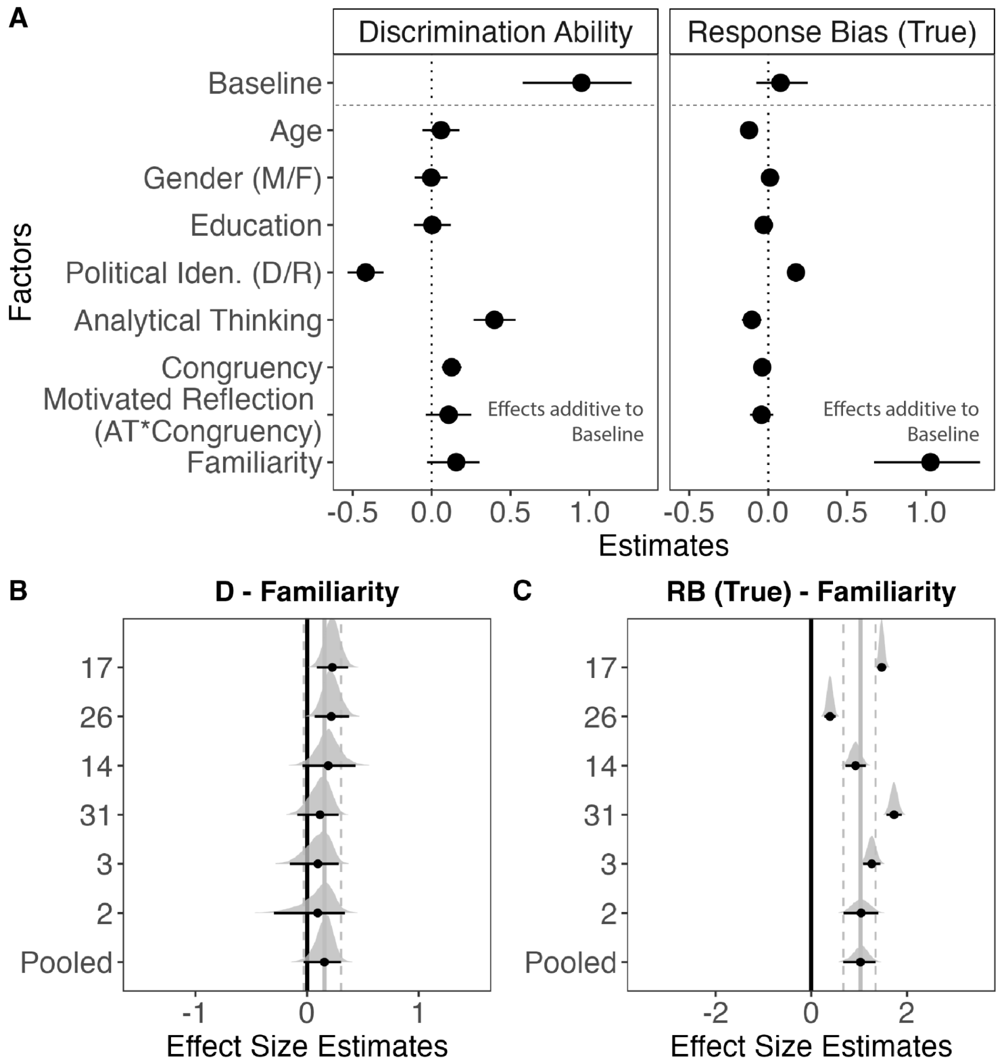

Figure S7. *Panel A: Complete-case SDT model estimates for familiarity. All results derive from a single SDT analysis using participants' responses (false news or true news) as the response variable but are shown in two panels to ease interpretation. Panel B and Panel C: Study-level SDT estimates for discrimination ability and response bias for familiarity. All estimates derive from a complete-case SDT model for familiarity (A). The left panels show the estimates for discrimination ability, with more positive (negative) values indicating higher (lower) discrimination ability. The right panels show the estimates for response bias, with more positive (negative) values indicating a higher (lower) likelihood to judge headlines as true (false). Dots represent the mean and the error bars the 95% CI of the posterior distribution. Note that all factors were mean centred. In Panel B and Panel C, studies are ordered from highest to lowest effect of familiarity on discrimination ability, and study numbers correspond to study numbers in Table S2. Baseline: Overall estimate of discrimination ability (A, left panel) and response bias (A, right panel). Political Iden. (D/R) = political identity. Congruency = ideological congruency. AT*Congruency = interaction between Analytical Thinking and Congruency. D = discrimination ability. RB = response bias. Pooled: Overall estimate of discrimination ability (B) and response bias (C).*

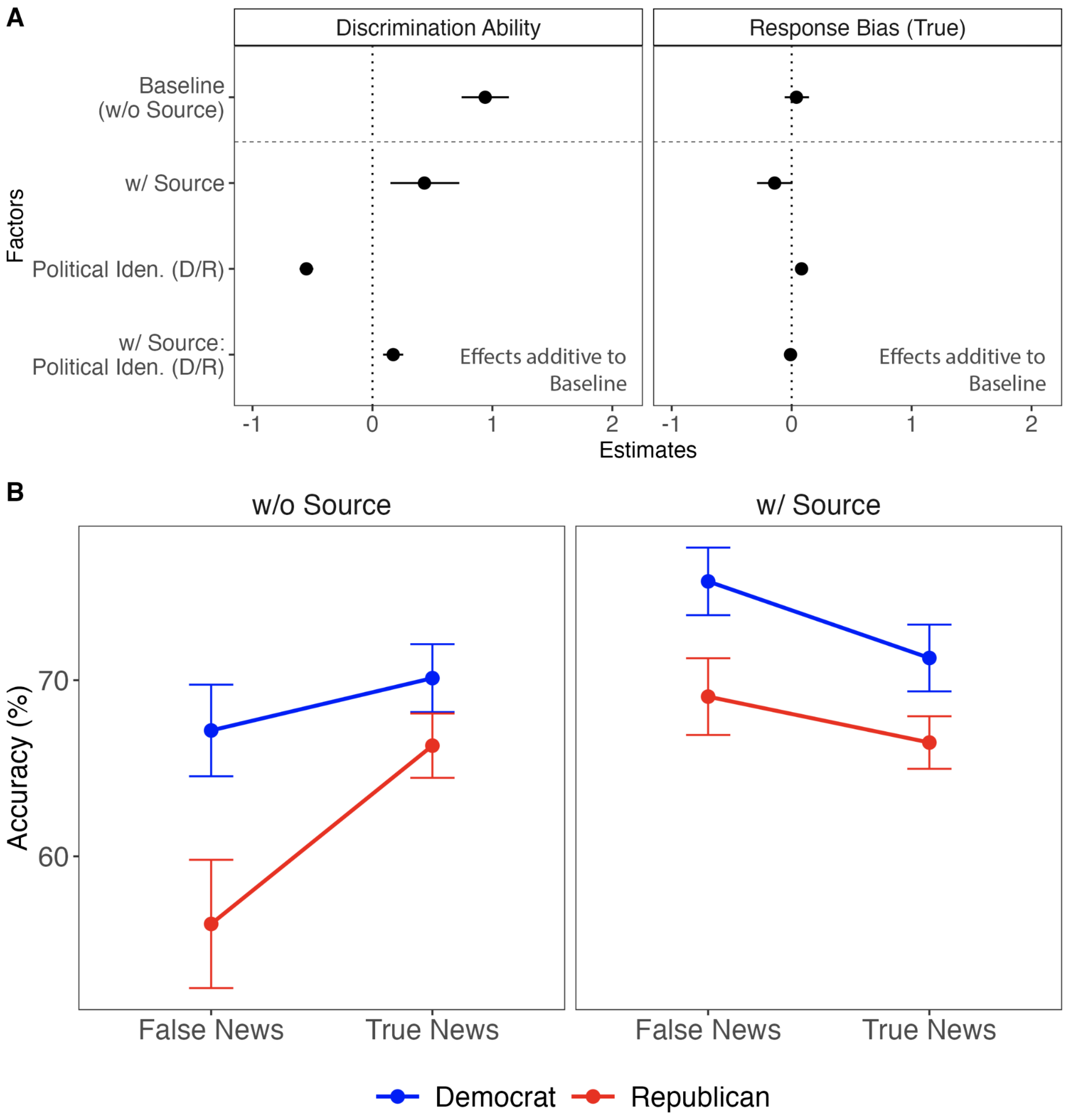

Figure S8. *Panel A: Signal detection theory model estimates for source display and political identity. The left panels show the estimates for discrimination ability, with more positive (negative) values indicating higher (lower) discrimination ability. The right panels show the estimates for response bias, with more positive (negative) values indicating a higher (lower) likelihood to judge headlines as true (false). Dots represent the mean; error bars represent the 95% credible interval (CI) of the posterior distribution. Baseline (w/o Source): Estimate of discrimination ability and response bias when headlines without source display are set as model baseline. w/o = without. w = with. Political Iden. (D/R) = political identity, coded Democrat to Republican and mean centred. Panel B: Accuracy for true and false news headlines for source display split by political identity. Lines connect accuracy scores between true and false news headlines across studies. Dots represent the aggregate mean with standard errors.*

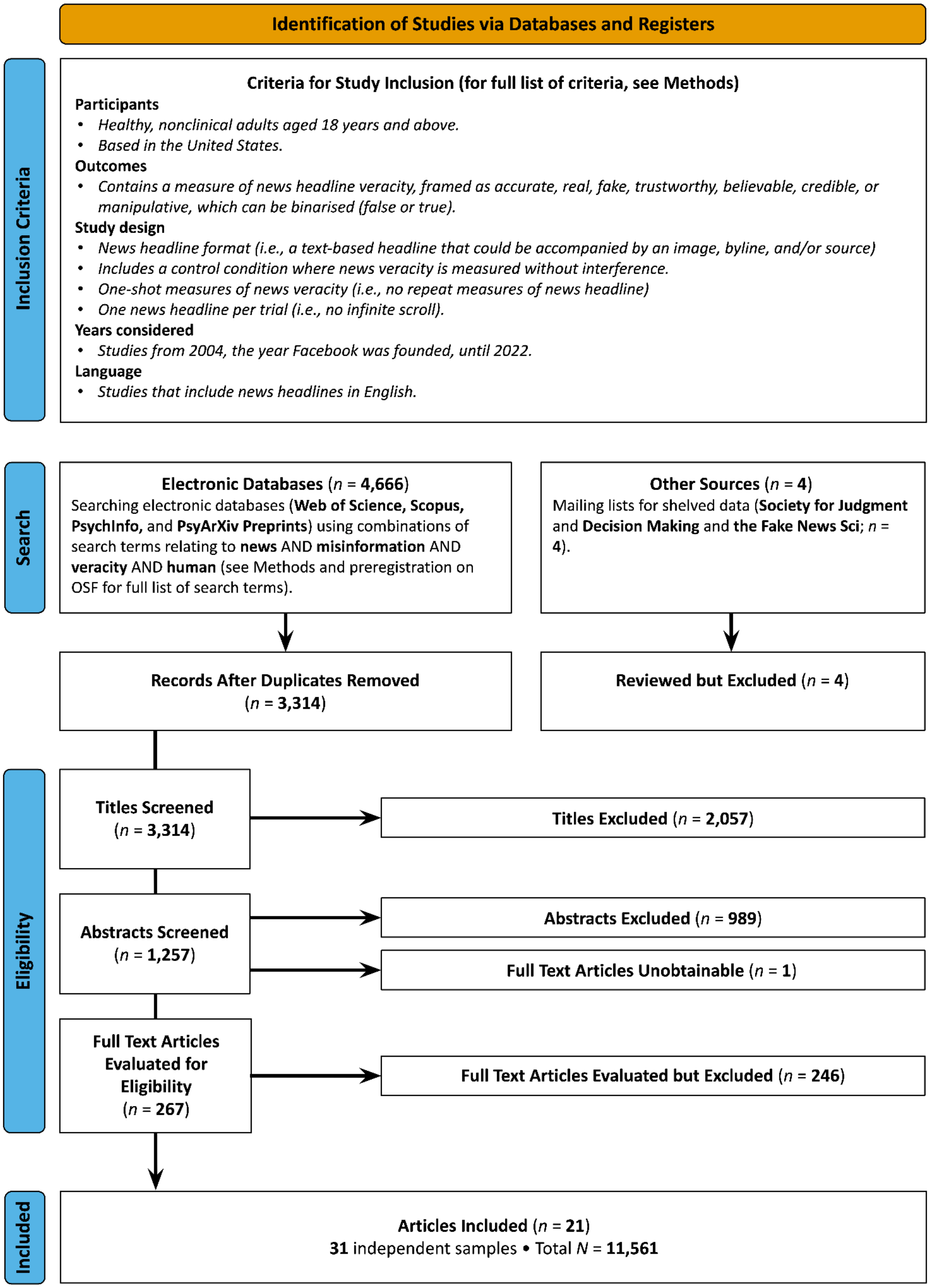

Figure S9. *PRISMA flow diagram summarising the study screening process.*

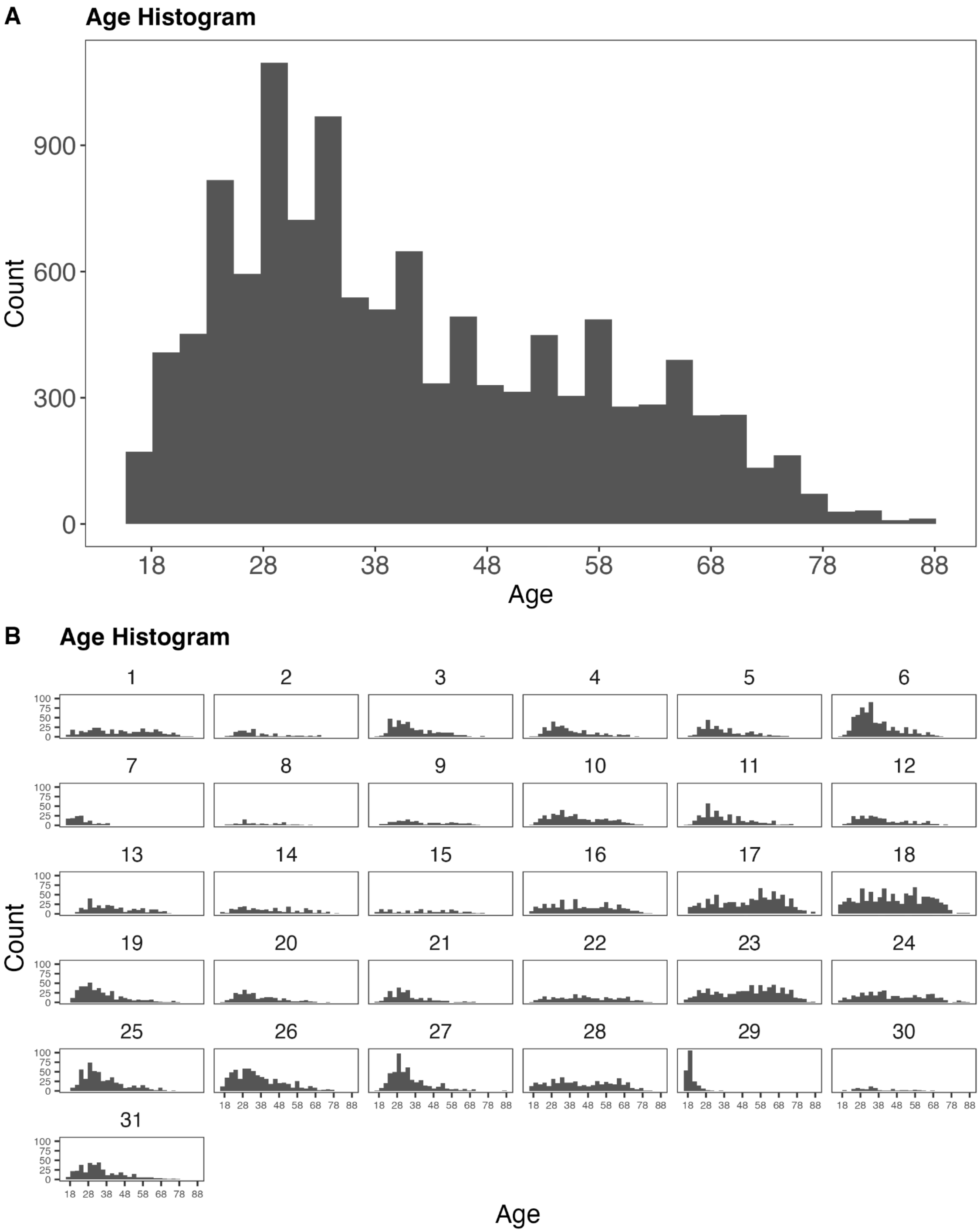

Figure S10. *Histogram for age across all studies (A) and separated by study (B). The study numbers correspond to study numbers in Table S2.*

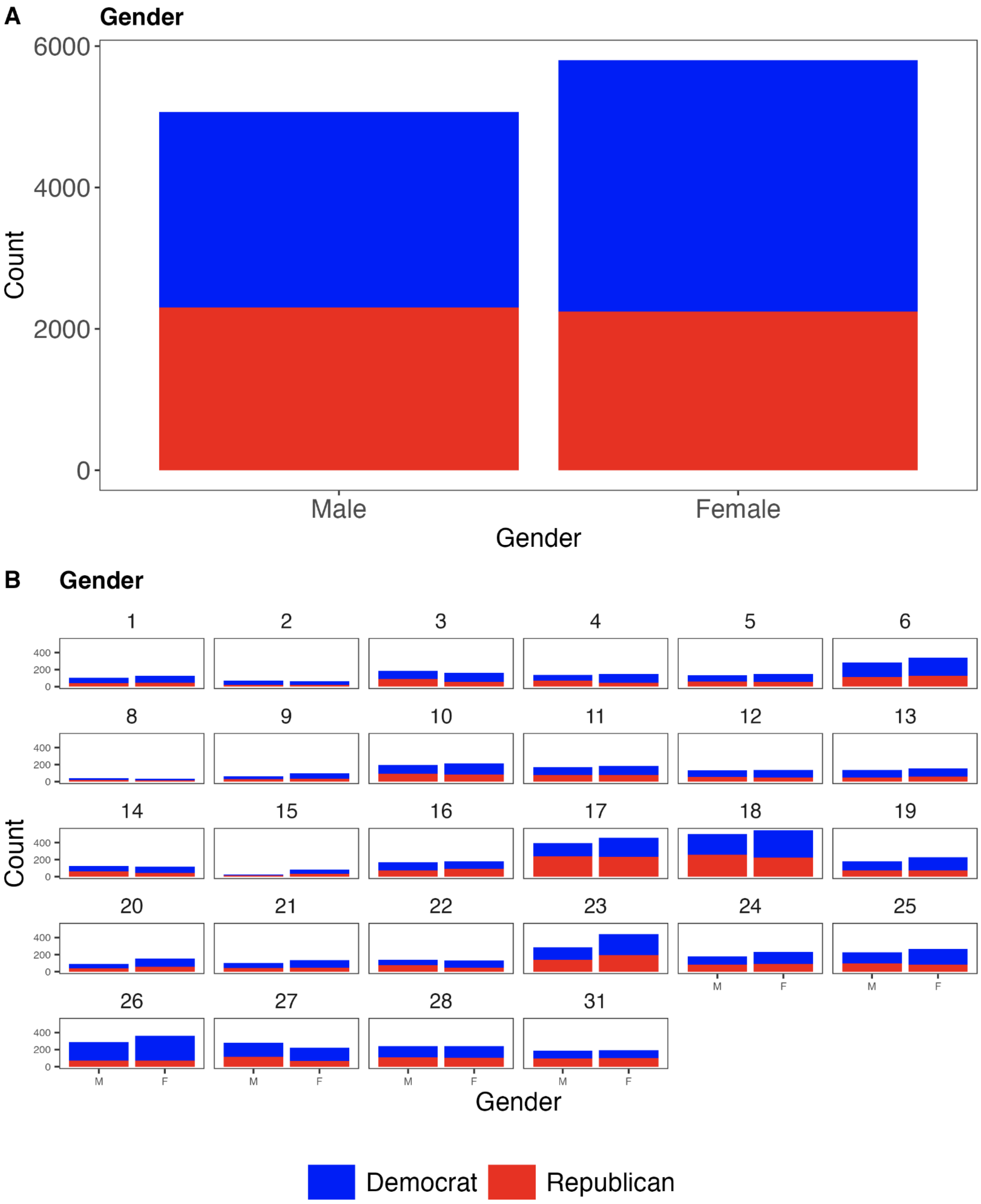

Figure S11. *Histogram for gender grouped by political identity across all studies (A) and separated by study (B). The study numbers correspond to study numbers in Table S2. M: Male. F: Female.*

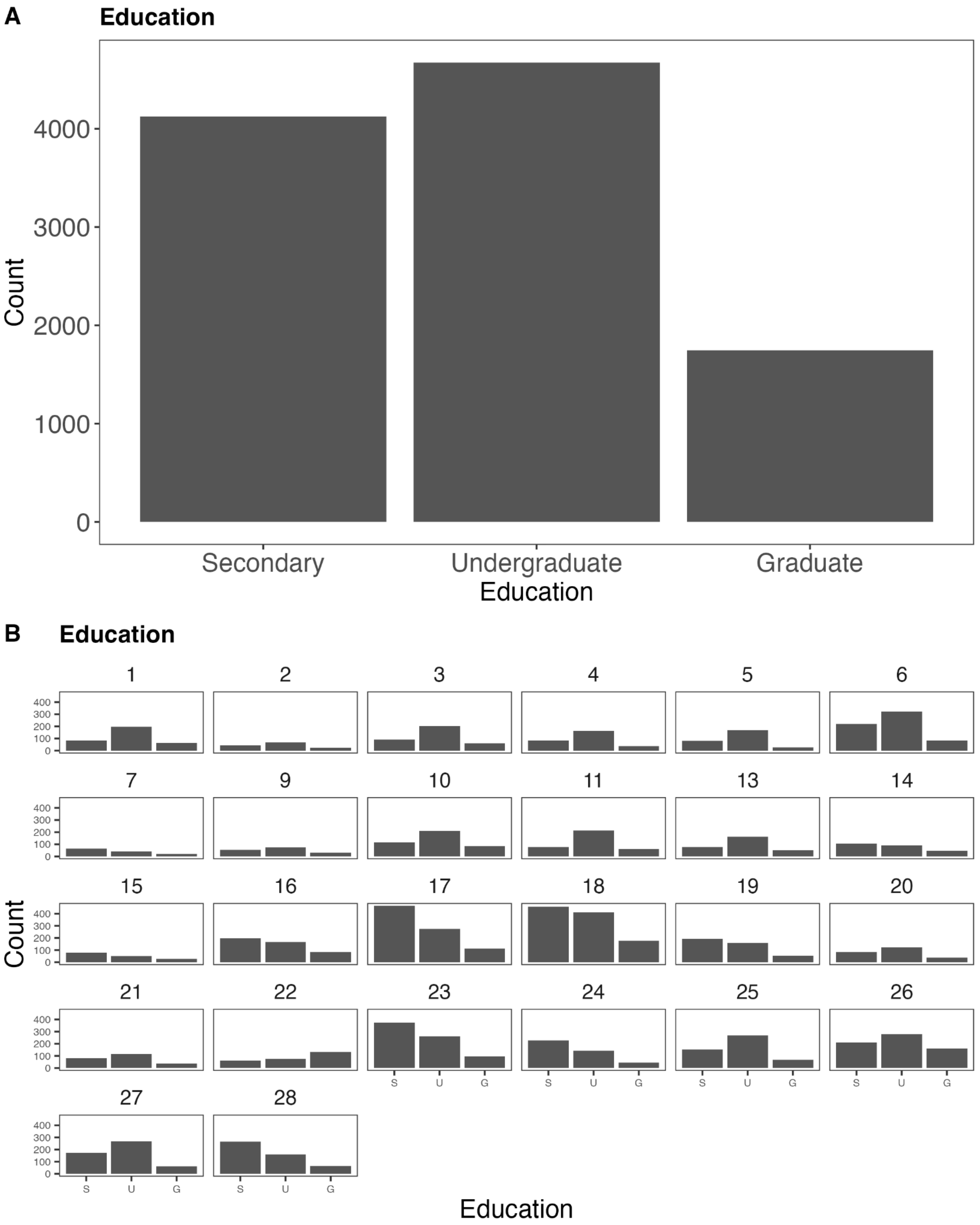

Figure S12. *Histogram for education across all studies (A) and separated by study (B). The study numbers correspond to study numbers in Table S2. S: Secondary. U: Undergraduate. G: Graduate.*

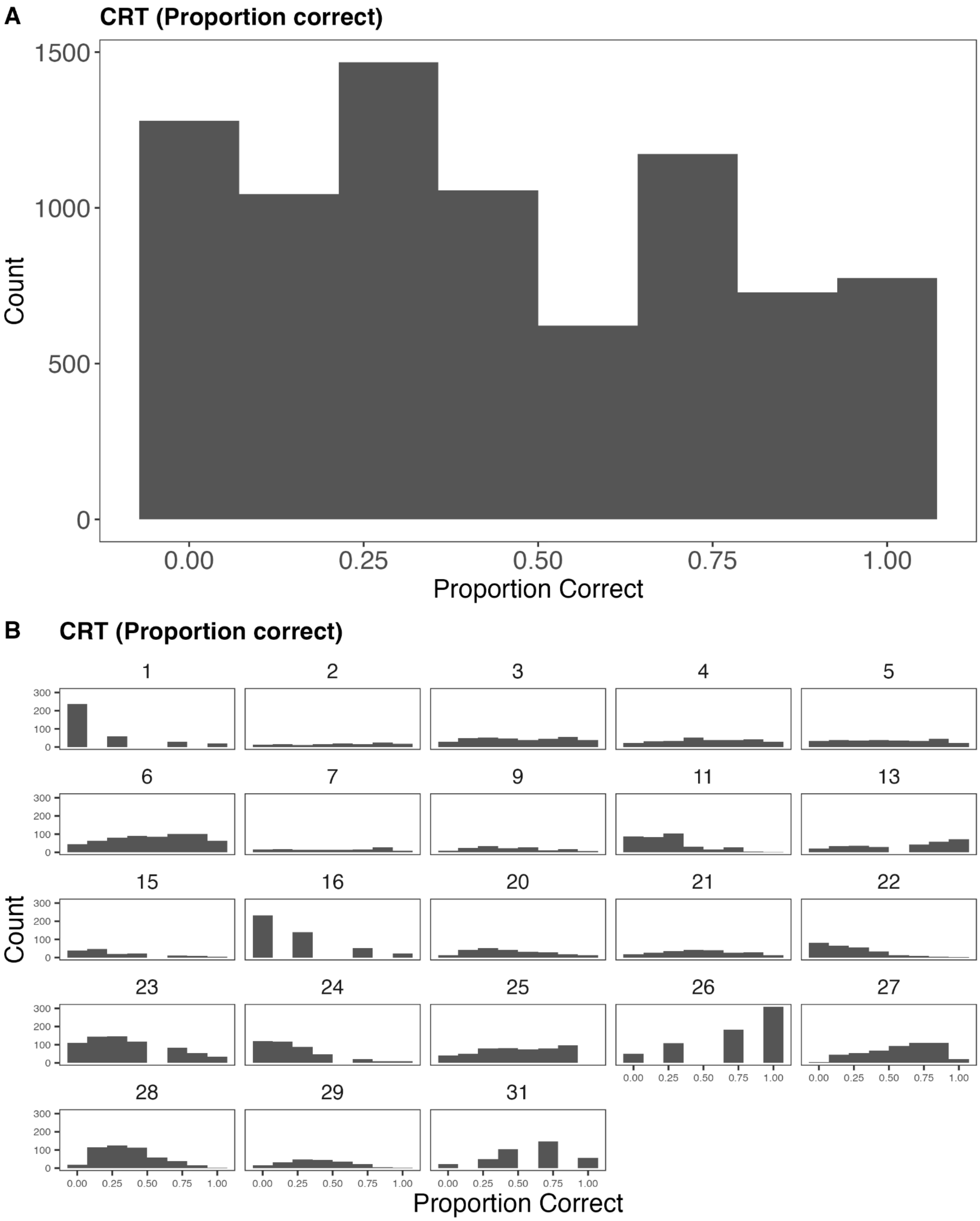

Figure S13. *Histogram for CRT proportion scores across all studies (A) and separated by study (B). The study numbers correspond to study numbers in Table S2.*

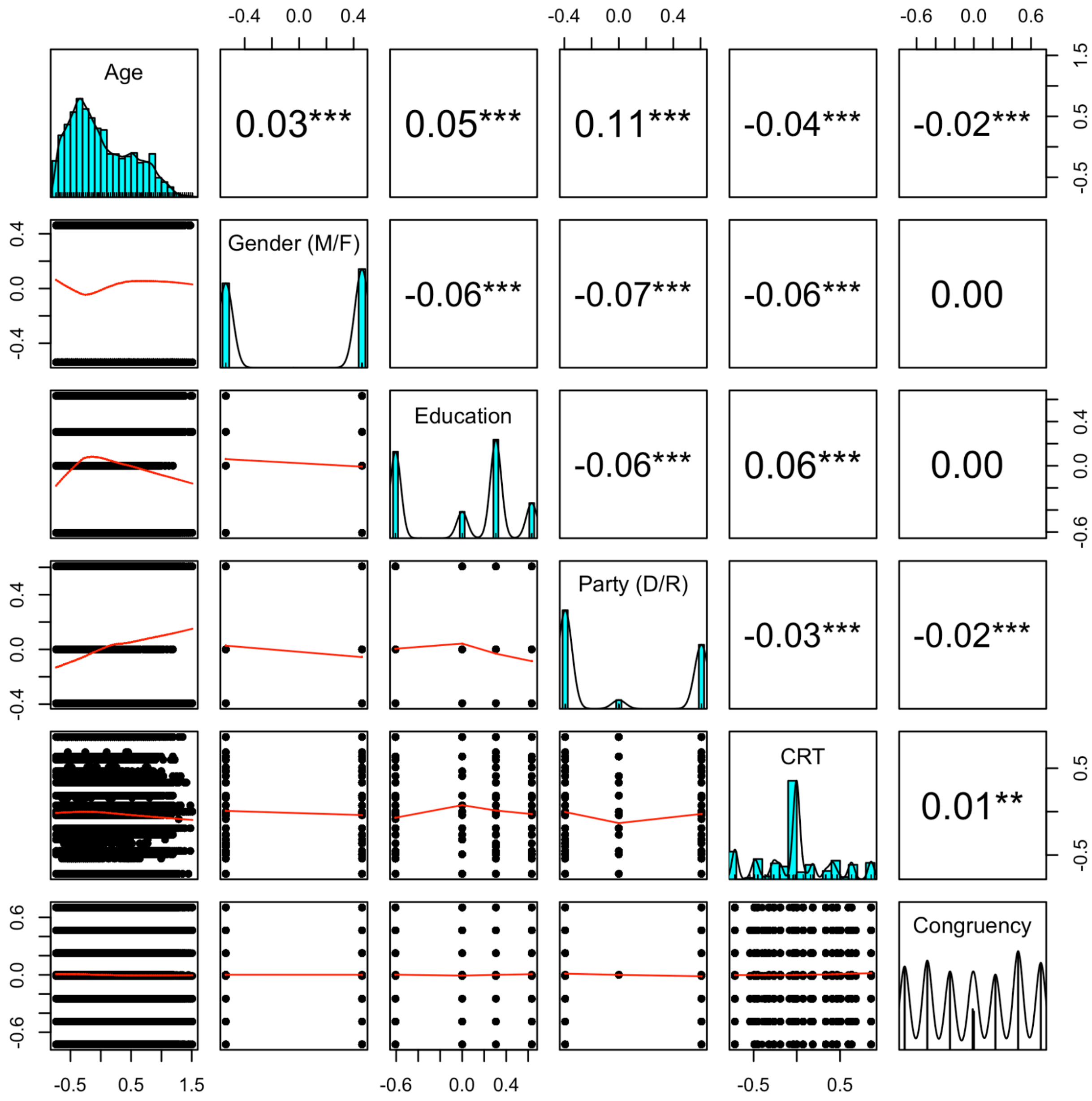

Figure S14. *Correlations between the demographic and psychological factors. The diagonal shows histograms of each of the factors. The lower triangle visualises their associations. The upper triangle depicts the Pearson correlation coefficients. The factors were all very weakly correlated, with the strongest association being with age and political identity (0.11). Gender (M/F) = coded Male to Female. Political Iden. (D/R) = political identity, coded Democrat to Republican. Congruency = ideological congruency.*

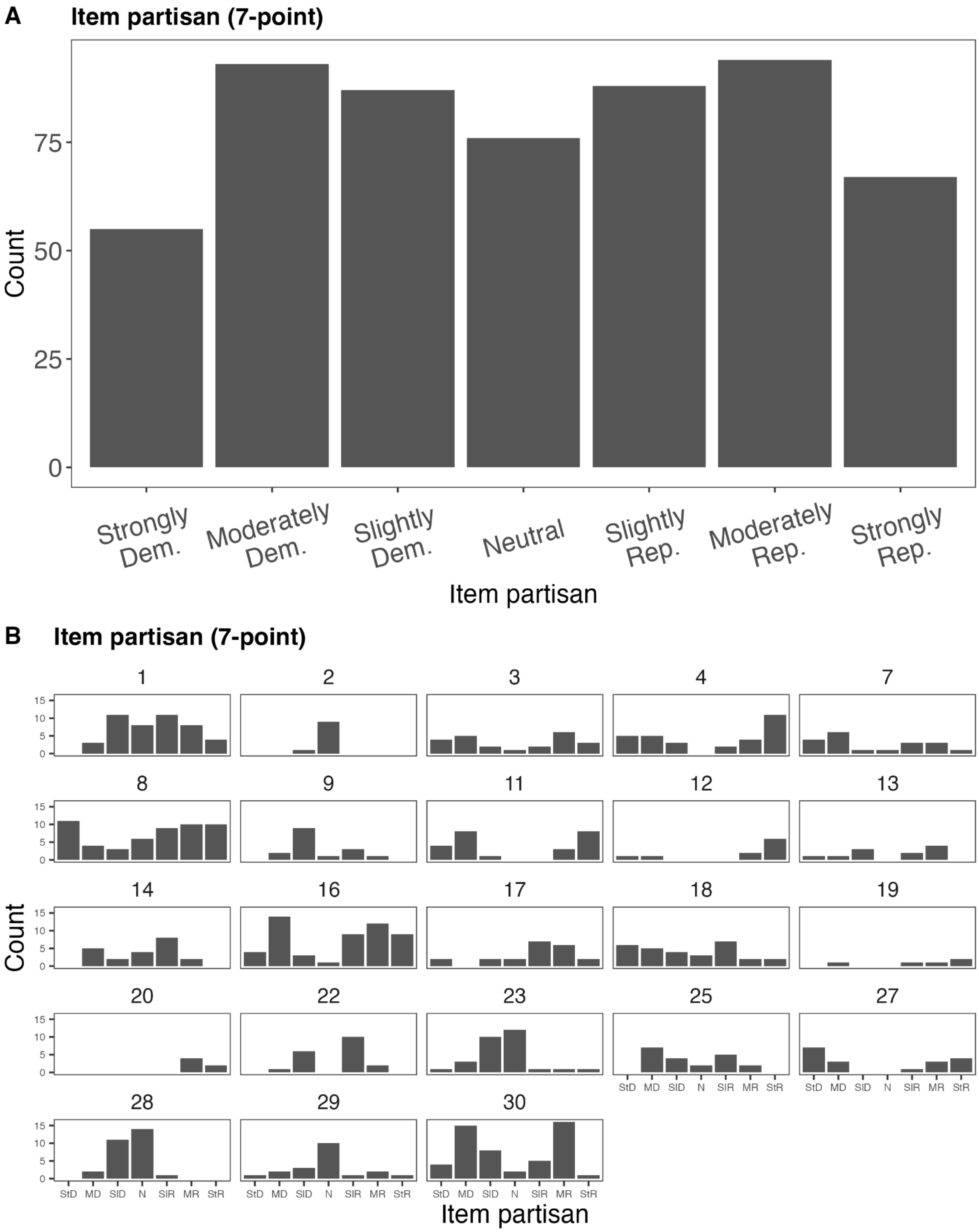

Figure S15. *Histogram for partisan leanings of news headlines across all studies (A) and separated by study. The study numbers correspond to study numbers in Table S2. Dem: Democrat. Rep: Republican. StD: Strongly Democrat. MD: Moderately Democrat. SlD: Slightly Democrat. N: Neutral. SlD: Slightly Republican. MD: Moderately Republican. StD: Strongly Republican.*

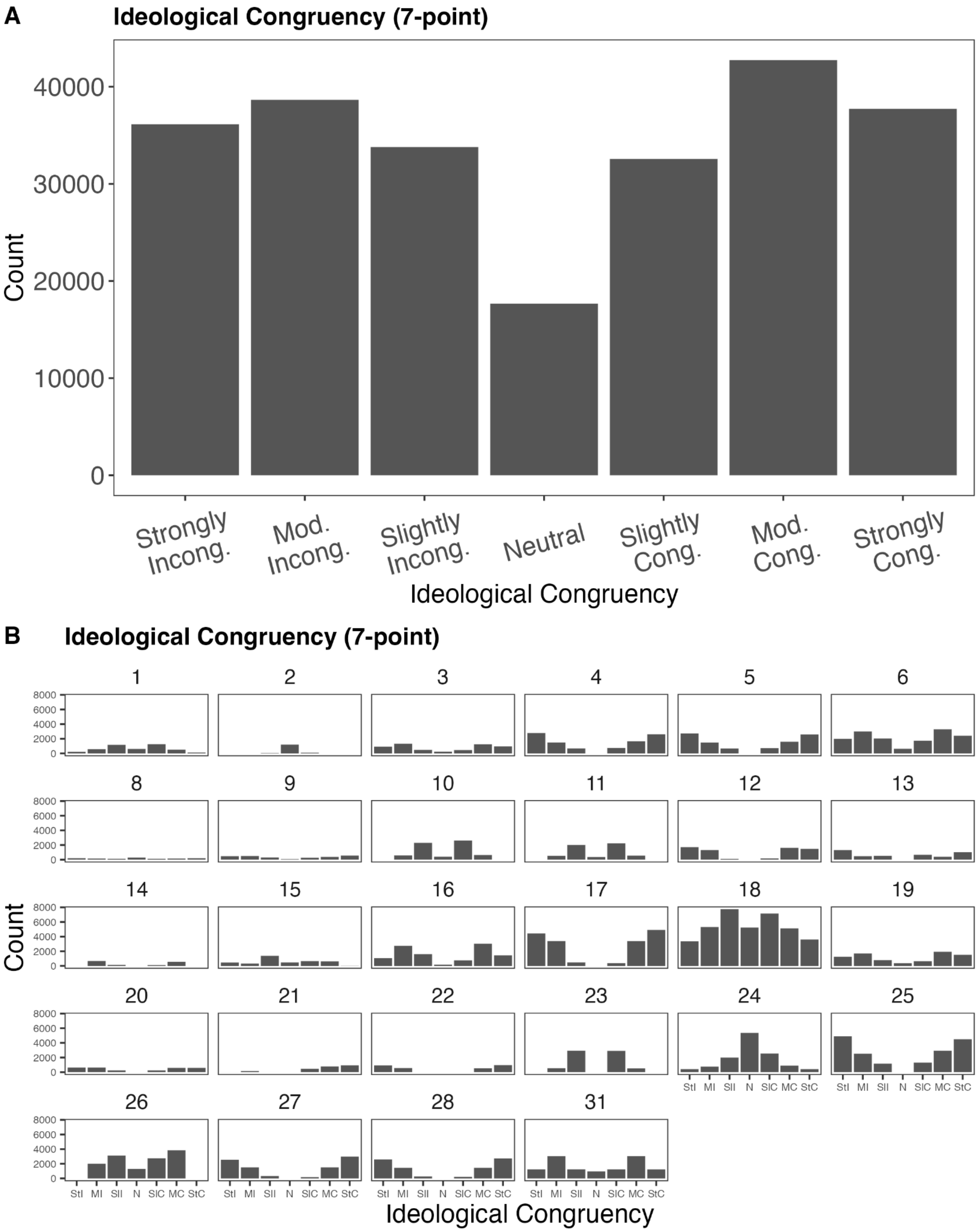

Figure S16. *Histogram for ideological congruence (7-point) across all studies (A) and separated by study. The study numbers correspond to study numbers in Table S2. Incon: Incongruent. Cong: Congruent. Mod: Moderately. StI: Strongly Incongruent. MI: Moderately Incongruent. SlI: Slightly Incongruent. N: Neutral. SlC: Slightly Congruent. MC: Moderately Congruent. StC: Strongly Congruent.*

Table S1. *Search terms for Web of Science, Scopus, and PsycINFO. For Web of Science and Scopus, to be indexed, studies needed to mention at least one term from each category of interest (i.e., news AND misinformation AND veracity AND human). For PsycINFO, to be indexed, studies needed to mention at least one term from each category (i.e., subject headings AND custom terms AND custom terms). Custom terms refer to the search class ".tw.", which includes the following: words that appear in the table of contents, title, abstract, and key concepts.*

Web of Science and Scopus categories

News	Misinformation	Veracity	Human
news	misinfo*	veracity	Men OR women*
headline*	disinfo*	accura*	male* OR female*
	fake*	real	adult*
	false	trustworthy	subject*
		credib*	participant*
		manipulative	individual*
		correct*	Democrat* OR Republican*
		true	Liberal* OR Conservative*
		susceptib*	"United States" OR "United States of America" OR US OR USA OR U.S. OR U.S.A.
		discern*	

PsycINFO categories

Subject headings	Custom terms	Custom terms
faking	misinformation	veracity
news media	fake news	accura*
information	fact-checking	real
social media	correction	trustworthy

messages	news media	credib*
exposure		manipulative
COVID-19		correct*
coronavirus		true
truth		susceptib*
false beliefs		discern*
skepticism		
political issues		

Table S2. *Overview of studies*

Reference	Study ID (Platform)	$N_{participants}$	$N_{headlines}$ (Total)*
Arechar et al. (2023). Understanding and combatting misinformation across 16 countries on six continents. *Nat. Hum. Behav.*	1 (Lucid)	352	20 (45)
Bago, B., Rand, D. G., & Pennycook, G. (2020). Fake news, fast and slow: Deliberation reduces belief in false (but not true) news headlines. *J. Exp. Psychol. Gen.*	2 (MTurk)	134	10
	3 (MTurk)	355	16 (24)
Brashier, N. M., Pennycook, G., Berinsky, A. J., & Rand, D. G. (2021). Timing matters when correcting fake news. *PNAS*.	4 (MTurk)	284	36
	5 (MTurk)	279	36
Bronstein, M. V., Pennycook, G., Bear, A., Rand, D. G., & Cannon, T. D. (2019). Belief in fake news is associated with delusionality, dogmatism, religious fundamentalism, and reduced analytic thinking. *J. Appl. Res. Mem. Cogn.*	6 (MTurk)	629	24
Bronstein, M. V., Pennycook, G., Buonomano, L., & Cannon, T. D. (2021). Belief in fake news, responsiveness to cognitive conflict, and analytic reasoning engagement. *Think. Reason.*	7 (Lab)	126	24
Calvillo, D. P., & Smelter, T. J. (2020). An initial accuracy focus reduces the effect of prior exposure on perceived accuracy of news headlines. *Cogn. Res. Princ. Implic.*	8 (MTurk)	75	16 (32)
	9 (MTurk)	159	16 (32)
Calvillo, D. P., Ross, B. J., Garcia, R. J. B., Smelter, T. J., & Rutchick, A. M. (2020). Political ideology predicts perceptions of the threat of COVID-19 (and susceptibility to fake news about it). *Soc. Psychol. Pers. Sci.*	10 (MTurk)	409	16
	11 (MTurk)	354	16

Calvillo, D. P., Garcia, R. J. B., Bertrand, K., & Mayers, T. A. (2021). Personality factors and self-reported political news consumption predict susceptibility to political fake news. *Pers. Individ. Dif.*	12 (MTurk)	266	24
Calvillo, D. P., Rutchick, A. M., & Garcia, R. J. B. (2021). Individual differences in belief in fake news about election fraud after the 2020 U.S. election. *Behav. Sci.*	13 (MTurk)**	291	15
Carnahan, D., Bergan, D. E., & Lee, S. (2021). Do corrective effects last? Results from a longitudinal experiment on beliefs toward immigration in the U.S. *Polit. Behav.*	14 (RN/SSI)**	247	6
Epstein, Z., Sirlin, N., Arechar, A., Pennycook, G., & Rand, D. (2023). The social media context interferes with truth discernment. *Sci. Adv.*	15 (Lucid)**	156	25
	16 (Lucid)	451	24 (60)
Garrett, R. K., & Bond, R. M. (2021). Conservatives' susceptibility to political misperceptions. *Sci. Adv.*	17 (YouGov)	852	20[†]
Longoni, C., Fradkin, A., Cian, L., & Pennycook, G. (2022). News from generative artificial intelligence is believed less. *2022 ACM Conf. Fairness, Accountability, and Transparency.*	18 (Lucid)	1047	42
Martel, C., Pennycook, G., & Rand, D. G. (2020). Reliance on emotion promotes belief in fake news. *Cogn. Res. Princ. Implic.*	19 (MTurk)	409	20 (32)
	20 (MTurk)	244	12
	21 (MTurk)	234	10 (20)
	22 (Lucid)	269	12
Newton, C., Feeney, J., & Pennycook, G. (2023). On the disposition to think analytically: Four distinct intuitive-analytic thinking styles. *Pers. Soc. Psychol. Bull.*	23 (YouGov)	729	10 (20)
Pennycook, G., McPhetres, J., Zhang, Y., Lu, J. G., & Rand, D. G. (2020). Fighting COVID-19 misinformation on social media: Experimental evidence for a scalable accuracy-nudge intervention. *Psychol. Sci.*	24 (Lucid)	414	30
Pennycook, G., Epstein, Z., Mosleh, M., Arechar, A. A., Eckles, D., & Rand, D. G. (2021). Shifting attention to	25 (MTurk)	497	36

accuracy can reduce misinformation online. *Nature*.			
Roozenbeek, J., Maertens, R., Herzog, S. M., Geers, M., Kurvers, R., Sultan, M., & van der Linden, S. (2022). Susceptibility to misinformation is consistent across question framings and response modes and better explained by myside bias and partisanship than analytical thinking. *Judgm. Decis. Mak.*	26 (Prolific)	650	20
Ross, R. M., Rand, D. G., & Pennycook, G. (2021). Beyond 'fake news': Analytic thinking and the detection of false and hyperpartisan news headlines. *Judgm. Decis. Mak.*	27 (MTurk)	505	20
	28 (Lucid)	490	20
Smelter, T. J., & Calvillo, D. P. (2020). Pictures and repeated exposure increase perceived accuracy of news headlines. *Appl. Cogn. Psychol.*	29 (Lab)	209	28
	30 (MTurk)	64	20
Sultan, M., Tump, A. N., Geers, M., Lorenz-Spreen, P., Herzog, S. M., & Kurvers, R. H. J. M. (2022). Time pressure reduces misinformation discrimination ability but does not alter response bias. *Sci. Rep*.	31 (Prolific)	381	32 (64)
Total	31	11,561	774[§]

Note. * = Some studies sampled headlines from a larger pool, with pool sizes in brackets. ** = Unequal base rates of true and false news headlines. [†] = Study included 12 waves of headlines, of which we used only the first. § = Total number of headlines across the 31 studies. However, some studies used the same headlines, resulting in a total of 562 unique headlines. RN/SSI = Research Now/SSI. Study numbers correspond to study numbers listed in the Supplementary Materials.

Table S3. *List of extracted variables of interest*

Variable	Description
Study and participant	
paper_ref	Paper reference (DOI)
study_id	Study identification number (1 to n). Note that one paper reference can contain multiple study_ids.
study_year	Year study conducted
study_platform	Name of platform used to conduct the study (e.g., Prolific)
study_base_rate	Base rate of true and false news headlines (e.g., 50% true: 50% false)
study_treatment	The name of the study/treatment the data is extracted from (e.g., control, active control, veracity-only)
part_id	Participant identification number (1 to n)
base_rate_informed	Whether participants are informed of study base rate or not (i.e., yes, no, NA)
News headline	
item_id	News headline identification number (1 to n; unique if possible)
item_veracity	Binary of item_veracity (i.e., false, true)
item_type	Type of news headline (e.g., political, covid, health)
item_type_GPT	Type of news headline as generated by GPT4 (i.e., political, covid, health)
item_presentation	Presentation mode of headline (e.g., headline only, headline and image)
item_generation	How news headlines were generated (i.e., human vs. AI)
item_partisan	Political leaning of news headlines (i.e., Republican, Democratic)

item_partisan_GPT	Political leaning of news headlines as generated by (i.e., Strongly Republican, Moderately Republican, Lean Republican, Neutral, Lean Democratic, Moderately Democratic, Strongly Democratic)
Demographic	
part_age	Participant age in years (minimum 18)
part_gender	Participant gender (i.e., male, female)
part_education_raw	Participant education raw (e.g., high school to doctoral level)
part_education	Participant education (i.e., Secondary, Undergraduate, and Graduate) turned into ridit scores.
part_political_identity_raw	Participant political identity (e.g., Strong Democrat, Moderate Democrat, Lean Democrat, Lean Republican, Moderate Republican, Strong Republican)
part_political_identity_binary	Binary of part_political_identity (i.e., Democrat, Republican)
Psychological	
CRT_type	Type of CRT administered (e.g., numeric, non-numeric, mixed)
CRT_number_questions	Number of CRT questions asked and answered (%)
CRT_correct	Number of correct CRT responses
CRT	Proportion of correct CRT responses (0-1)
ideological_congruency	Whether the political leaning of news headline is congruent (incongruent) with the participants' political leaning (i.e., Strongly incongruent, Moderately incongruent, Lean incongruent, Neutral, Lean congruent, Moderately congruent, Strongly congruent)
familiarity_response_mode	Response mode of familiarity question (e.g., binary, 6-point Likert scale)
familiarity_response_raw	Familiarity response (e.g., unfamiliar, familiar)
familiarity_response_binary	Binary of familiarity_response_raw (i.e., unfamiliar, familiar)

Veracity judgment	
veracity_framing	Question framing of veracity question (e.g., real-fake, credible)
veracity_response_mode	Response mode of veracity question (e.g., binary, 6-point Likert scale)
veracity_response_raw	Participant response (e.g. false/true [for binary response mode] or 1-6 [for 6-point Likert scale])
veracity_response_binary	Binary of veracity_response_raw (i.e., false, true)

Table S4. *Aggregate descriptive statistics ($N_{participants}$ = 11,561)*

Variable	Descriptor	Value
Age	Mean (SD)	41.29 (15.68)
	Range	18 - 88
Gender (%)	Female (Male)	53.91 (46.09)
Education (%)	Secondary	39.17
	Undergraduate	44.31
	Graduate	16.57
Political identity (%)	Republican (Democrat)	41.85 (58.15)

Note: There is missing data across the following variables: Education (10.71%; 4/31 studies) and Political identity (6.52%; 3/31 studies).

Section S1: Re-coding Partisan Leaning of Headlines

To calculate the partisan leanings of each headline, the following two prompts were used and in the following order.

- Prompt 1: “You will be presented with headlines and will have to answer the following question regarding the headline: Assuming the headline is entirely accurate, how favourable is it to Democrats (liberals) or Republicans (conservatives)? In a forced choice manner, you can ONLY respond using these two options, either as Favourable for Democrats (democrat_leaning) or Favourable for Republicans (republican_leaning). Your response should strictly adhere to this binary choice (democrat_leaning or republican_leaning) without exceptions, even if the headline appears neutral or unrelated to US politics. In your answer, first provide the partisan leaning (democrat_leaning or republican_leaning). This should be separated by ‘<separate>’, which will follow your explanation. Here's the headline: [HEADLINE TEXT]”
- Prompt 2: “Now, answer the same question using the following seven options: either as Strongly Favourable for Democrats (Strongly Democrat), Moderately Favourable for Democrats (Moderately Democrat), Slightly Favourable for Democrats (Slightly Democrat), Neutral (Neutral), Slightly Favourable for Republicans (Slightly Republican), Moderately Favourable for Republicans (Moderately Republican), or Strongly Favourable for Republicans (Strongly Republican). Note that you can now respond with a ‘Neutral’, making it no longer a forced choice between democratic_leaning or republican_leaning. In your answer, first provide the partisan leaning (Strongly Democrat, Moderately Democrat, Slightly Democrat, Neutral, Slightly Republican, Moderately Republican, and Strongly Republican). This should be separated by ‘<separate>’, which will follow your explanation. Here's the headline: [HEADLINE TEXT].”

Note that the separator (“<separate>”) was used to distinguish between the categorisation and the explanation during data processing.

Section S2: Full Model Specification and Explanation

```
model. <- brm(sayTrue ~
                  isTrue*age + # age
                  isTrue*gender  + # gender
                  isTrue*education  + # education
                  isTrue*pol_party  + # political identity
                  isTrue*CRT + # analytical thinking
                  isTrue*congruency + # ideological congruency
                  isTrue*CRT*congruency + # motivated reflection
                  #isTrue*familiarity + # familiarity

                  (1 + isTrue | part_id) +
                  (1 + isTrue*age + isTrue*gender  + isTrue*education  + isTrue*pol_party  +
                  isTrue*CRT + isTrue*congruency + isTrue*CRT*congruency || study_id) +
                  (1 | item_id),

                data = dat_model_mean_impute,
                family = bernoulli(link = "probit"),
                init = 0,
                iter = 10000
)
```

Conducting a signal detection analysis (SDT) via a probit regression.

In SDT, we are interested in two parameters: discrimination ability and response bias. Both discrimination ability and response bias can be calculated with the hit rate and the false alarm rate.

- Hit Rate (H): This is the probability that a participant correctly identifies a true signal. In our context, it is the probability that a participant says a headline is true when it actually is true.
- False Alarm Rate (F): This is the probability that a participant incorrectly identifies a false signal as true. In our context, it is the probability that a participant says a headline is true when it is actually false.

Discrimination ability and response bias are defined as follows, respectively, where z refers to z-scores:

$d' = z(H) - z(F)$

$c = 1/2\,(z(H) + z(F))$[1]

Prior work has established that it is possible to infer both discrimination ability and response bias via a probit regression (see 1-3), such that $d' = \beta 1$ and that $c = \beta 0$ in a regression formula (see below). A simple regression formula would look as follows:

[1] Please note that it is a traditional convention that the criterion is negated but we do not make this distinction for the sake of clarity.

$sayTrue \sim Bernoulli(pi)$

$pi = \Phi(\beta 0 + \beta 1 \cdot isTrue)$

Where:

- sayTrue is the outcome variable (whether the participant said false or true)
- pi is the probability of saying true
- isTrue (whether the headline is false or true is the predictor variable (coded -0.5, 0.5)
- Φ represents the probit link function (which gives z-scores).

Discrimination ability

When isTrue = −0.5 (headline is false), the expression inside Φ becomes:

$\Phi(\beta 0 - 0.5\beta 1) = z(F)$

This is the same as $z(F)$, when participants say a headline is true when it is actually false.

When isTrue = 0.5 (headline is true), the expression inside Φ becomes:

$\Phi(\beta 0 + 0.5\beta 1) = z(H)$

This is the same as $z(H)$, when participants say a headline is true when it is actually true. This results in:

$\beta 1 = z(H) - z(F) = d'$

Response bias

Moving on to response bias, it is defined as the average of $z(H) + z(F)$:

$c = 1/2\,(z(H) + z(F))$

As β0 (the intercept) captures when isTrue is at its midpoint, it effectively is the average of the z-scores for the hit rate and the false alarm rate. This results in:

$\beta 0 = 1/2\,(z(H) + z(F)) = c$

Overall, β0 is the intercept in the regression model, representing the z-score (since the probit link function gives z-scores) of a participant saying a headline is true when isTrue is at its midpoint (0).

Adding a factor

In an example regression where we include analytical thinking (CRT), the regression formula would look as follows:

$sayTrue \sim Bernoulli(pi)$

$$pi = \Phi(\beta 0 + \beta 1 \cdot isTrue + \beta 2 \cdot CRT + \beta 3 \cdot isTrue \times CRT)$$

Where:

- As before, sayTrue is the outcome variable (whether the participant said false or true)
- pi is the probability of saying true
- isTrue (whether the headline is false or true; coded -0.5, 0.5)
- CRT (range 0-1, mean centred)

When examining the main effect of a single factor, such as CRT, the coefficient (β2) represents its influence on the response bias. This indicates a general bias in responding “true” or “false” that is consistent regardless of whether the statement is actually true. Given that we find a negative effect of CRT on response bias in our meta-analysis, this suggests that, on average, participants with higher CRT scores were more biased towards responding false compared to participants with lower CRT scores.

Considering the interaction effect (β3). This means that the ability to discriminate between true and false headlines changes depending on the level of CRT. We found a positive interaction term, this indicates that participants with higher CRT scores were better at distinguishing between true and false headlines.

We recommend interested readers pursue the referenced papers in the main text (see 1-3).

SI References

1. LT DeCarlo, Signal detection theory and generalized linear models. *Psychol. Methods* **3**, 186–205 (1998).
2. JN Rouder, J Lu, An introduction to Bayesian hierarchical models with an application in the theory of signal detection. *Psychon. Bull. & Rev*. **12**, 573–604 (2005).
3. M Vuorre, Bayesian estimation of signal detection models (https://mvuorre.github.io/posts/2017-10-09-bayesian-estimation-of-signal-detection-theory-models/) (2017).
